# Phosphorylation-induced changes in the PDZ domain of Dishevelled 3

**DOI:** 10.1038/s41598-020-79398-5

**Published:** 2021-01-15

**Authors:** Miroslav Jurásek, Jitender Kumar, Petra Paclíková, Alka Kumari, Konstantinos Tripsianes, Vítězslav Bryja, Robert Vácha

**Affiliations:** 1 National Centre for Biomolecular Research, Faculty of Science, Masaryk University, Kamenice 753/5, 625 00 Brno, Czech Republic; 2 CEITEC – Central European Institute of Technology, Masaryk University, Kamenice 753/5, 625 00 Brno, Czech Republic; 3 Department of Experimental Biology, Faculty of Science, Masaryk University, Brno, 62500 Czech Republic; 4 Institute of Biophysics, Academy of Sciences of the Czech Republic, v.v.i., Brno, 612 65 Czech Republic

**Keywords:** Computational biophysics, Molecular conformation

## Abstract

The PDZ domain of Dishevelled 3 protein belongs to a highly abundant protein recognition motif which typically binds short C-terminal peptides. The affinity of the PDZ towards the peptides could be fine-tuned by a variety of post-translation modifications including phosphorylation. However, how phosphorylations affect the PDZ structure and its interactions with ligands remains elusive. Combining molecular dynamics simulations, NMR titration, and biological experiments, we explored the role of previously reported phosphorylation sites and their mimetics in the Dishevelled PDZ domain. Our observations suggest three major roles for phosphorylations: (1) acting as an on/off PDZ binding switch, (2) allosterically affecting the binding groove, and (3) influencing the secondary binding site. Our simulations indicated that mimetics had similar but weaker effects, and the effects of distinct sites were non-additive. This study provides insight into the Dishevelled regulation by PDZ phosphorylation. Furthermore, the observed effects could be used to elucidate the regulation mechanisms in other PDZ domains.

## Introduction

Protein-protein interactions play a crucial role in many biological processes. The PDZ domain^1^ is a well-established protein-protein recognition motif with moderate to high affinity^2^ that can be found in around 270 different instances within the human proteome^3^ and is essential in cell trafficking, scaffolding, and signal transduction^4^. PDZ can bind a number of various partners ranging from short C-terminal peptides through intramolecular motifs^5,6^ to lipids^7–10^. PDZ is a relatively small 80–100-residues-long domain, composed of six $\beta$-stranded $\beta$ sandwiches with two $\alpha$ helices. The binding site is situated in a groove between the $\beta 2$ strand and $\alpha 2$ helix. The groove is terminated by a carboxylate-binding loop that is responsible for the hydrogen bonding of the ligand carboxyl group^11^. The affinity of PDZ towards various ligands can be dynamically modulated by disulfide bond formation^12^ and post-translation modifications such as methylation^13^ or phosphorylation^14,15^,which can be present on both the ligand^16–19^ and the PDZ^20–25^. However, a molecular understanding of how the affinities are modulated by phosphorylation and what the associated structural changes are remains elusive^26^.

The versatile binding properties of PDZ are typically exploited in signaling hubs and scaffolding proteins^4,27,28^ such as a Dishevelled protein (DVL), a key component of Wnt signaling pathways^29^. Upon Wnt pathway activation, DVL is heavily phosphorylated by multiple different kinases, of which the most important are casein kinase 1 (CK1) and NEK2. Our unbiased proteomic screen in cells^30,31^ has recently shown that the PDZ of DVL is phosphorylated at four residues in a kinase-specific pattern. Moreover, we showed that two out of these four phosphorylation sites in DVL PDZ could modulate PDZ affinity towards the DVLs C-terminus, which is associated with an auto-inhibitory function^32^. PDZ phosphorylation sites are conserved in all metazoa; it is therefore anticipated that the PDZ phosphorylation code is essential for DVL’s function and the modulation of its interactions with downstream effectors. However, how PDZ phosphorylation sites and/or their combinations affect the structure and binding affinity of PDZ to cognate ligands is unknown.

**Figure 1:**
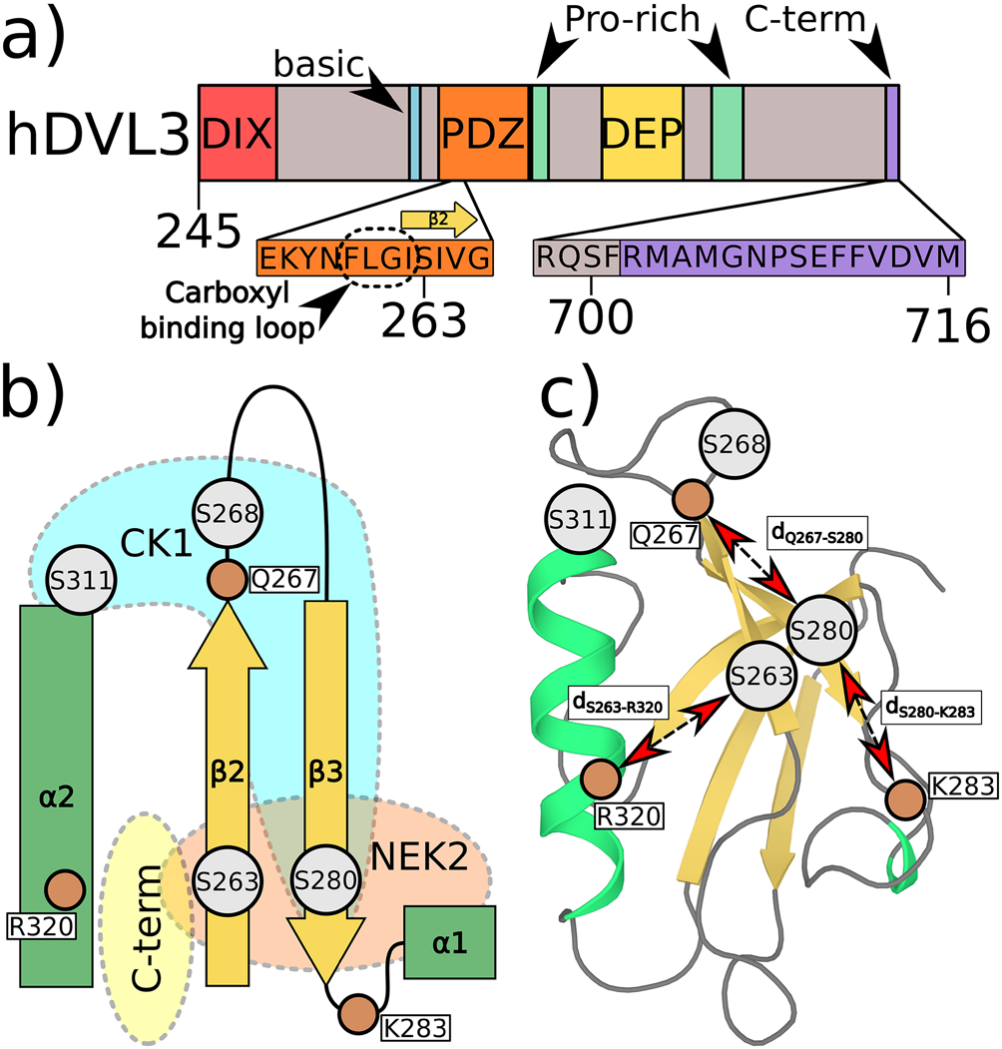
(**a**) Primary structure of the hDVL3 protein with position of PDZ and other domains/regions depicted. Amino acid sequences are shown for the C-term binding site and the C-terminus. (**b**) Schematic representation of the PDZ domain and its C-term binding site. The helix $\alpha 2$ and two $\beta$-sheets the $\beta 2$ and the $\beta 3$ are shown. Positions of four phosphorylations sites and important residues interacting with phospho/mimetic variants are depicted with gray and brown circles, respectively. (**c**) Homology model of the hDVL3 PDZ domain based on the structure of the hDVL2 structure (PDB 2rey) with a secondary structure assignment based on the PDB 2rey. Distances between phosphorylation sites and important residues are depicted.

Here, we set out to study the aforementioned phosphorylation sites of the PDZ from the third isoform of human DVL (hDVL3)(see Fig. 1). We used an all-atom molecular dynamics (MD) simulation to investigate the structural changes induced in the PDZ domain by individual phosphorylations at S263, S268, S280, and S311 residues or combinations thereof. The MD simulations allowed us to study the effects of specific phosphorylation states which are difficult to reach with experiments^33,34^. Moreover, simulations allowed us to directly compare phosphorylations to their corresponding phospho-mimetic mutants, commonly used in experiments. The key findings were validated by nuclear magnetic resonance (NMR) spectroscopy and biological assays.

## Results

### Dynamics of PDZ wt

**Figure 2:**
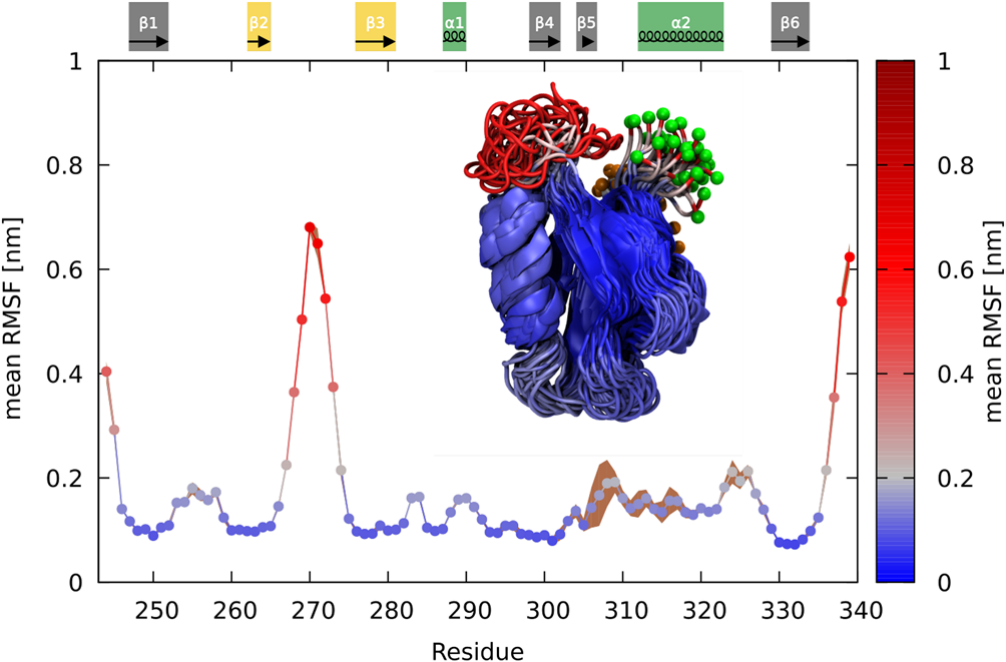
The PDZ wt domain mean Root Mean Square Fluctuation (RMSF). Values are derived from two independent $1\upmu \hbox {s}$ long simulations. RMSF standard deviation is displayed in brown color. Secondary structure elements are highlighted above the graph (elements involved in phosphorylations are colored). Inlet corresponds to the aligned trajectory of the PDZ wt, with snapshots separated by 25 ns. Coloring corresponds to mean RMSF values in the graph. N-terminus and C-terminus are highlighted with orange and green spheres, respectively.

A general trend in our simulations was that the PDZ domain remained relatively stable, with only loops and extensions being significantly mobile. The highest Root Mean Square Fluctuation (RMSF) was observed for 10 residues in the long loop between strands $\beta 2$ and $\beta 3$ ($\beta 2$–$\beta 3$) and the domain termini. Slightly increased fluctuations were observed for the short $\alpha 1$ helix, and a region around the binding site that engages the free carboxyl group of the ligand through hydrogen bonding (the C-terminal region of the $\alpha 2$ helix and the N-terminal part of the $\beta 2$ strand, see Fig. 2). RMSD analysis provided three distinct clusters of PDZ conformations corresponding to three distinct conformations of the long loop: The relative orientations of the loop were close to the $\alpha 2$ helix, close to PDZ termini or in between the two (see Fig. S2). The short $\alpha 1$ helix underwent fast unfolding/folding transitions (see Fig. S3) and it took $1\,\upmu \hbox {s}$ to converge the data, including the RMSF profiles, from two independent simulations (see Fig. 2).

**Figure 3:**
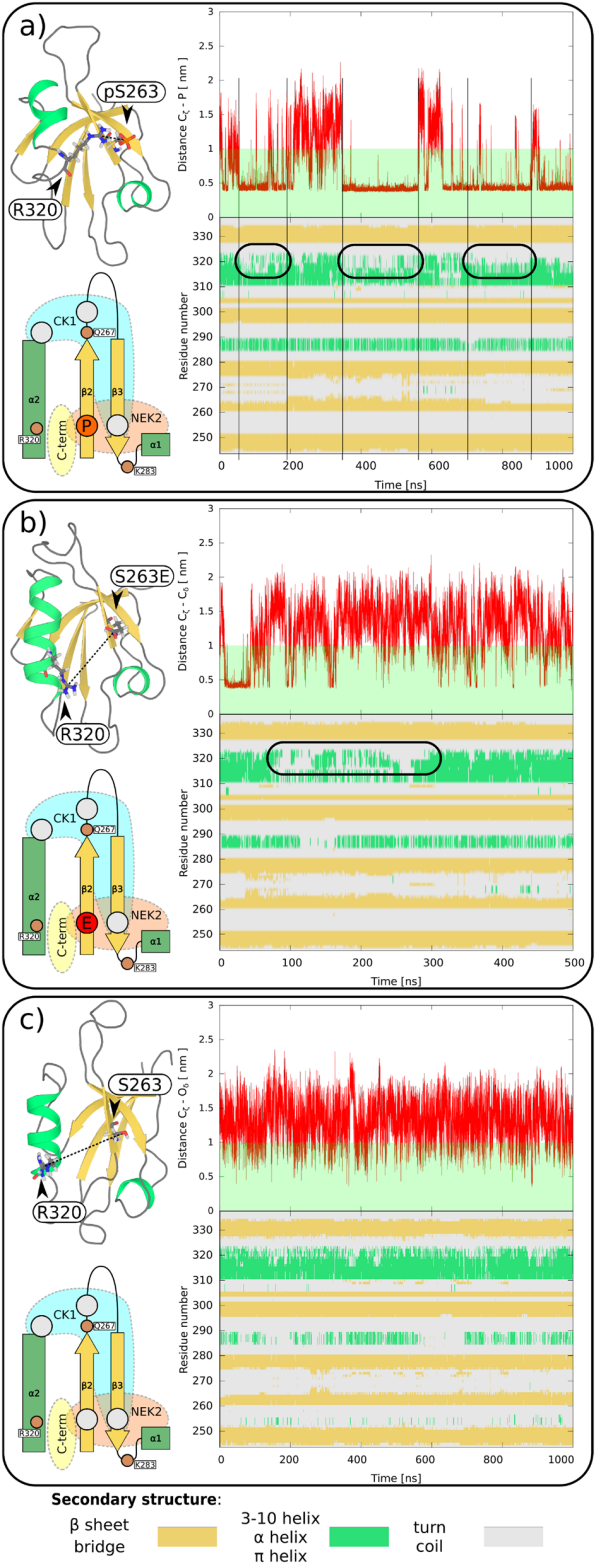
Effects of (**a**) the phosphoserine pS263 and (**b**) the phospho mimetic S263E on the PDZ structure, (**c**) show interactions in wt for comparison. In each panel: top left—system snapshot; bottom left—schematic representation; top right—time evolution of the distance between $\hbox {C}_{\zeta }$ atom in R320 and P/$\hbox {C}_\delta$/$\hbox {O}_\delta$ atoms in pS263/S263E/wt, respectively; and bottom right—the time evolution of the PDZ secondary structure (color coding: below the picture).

**Figure 4:**
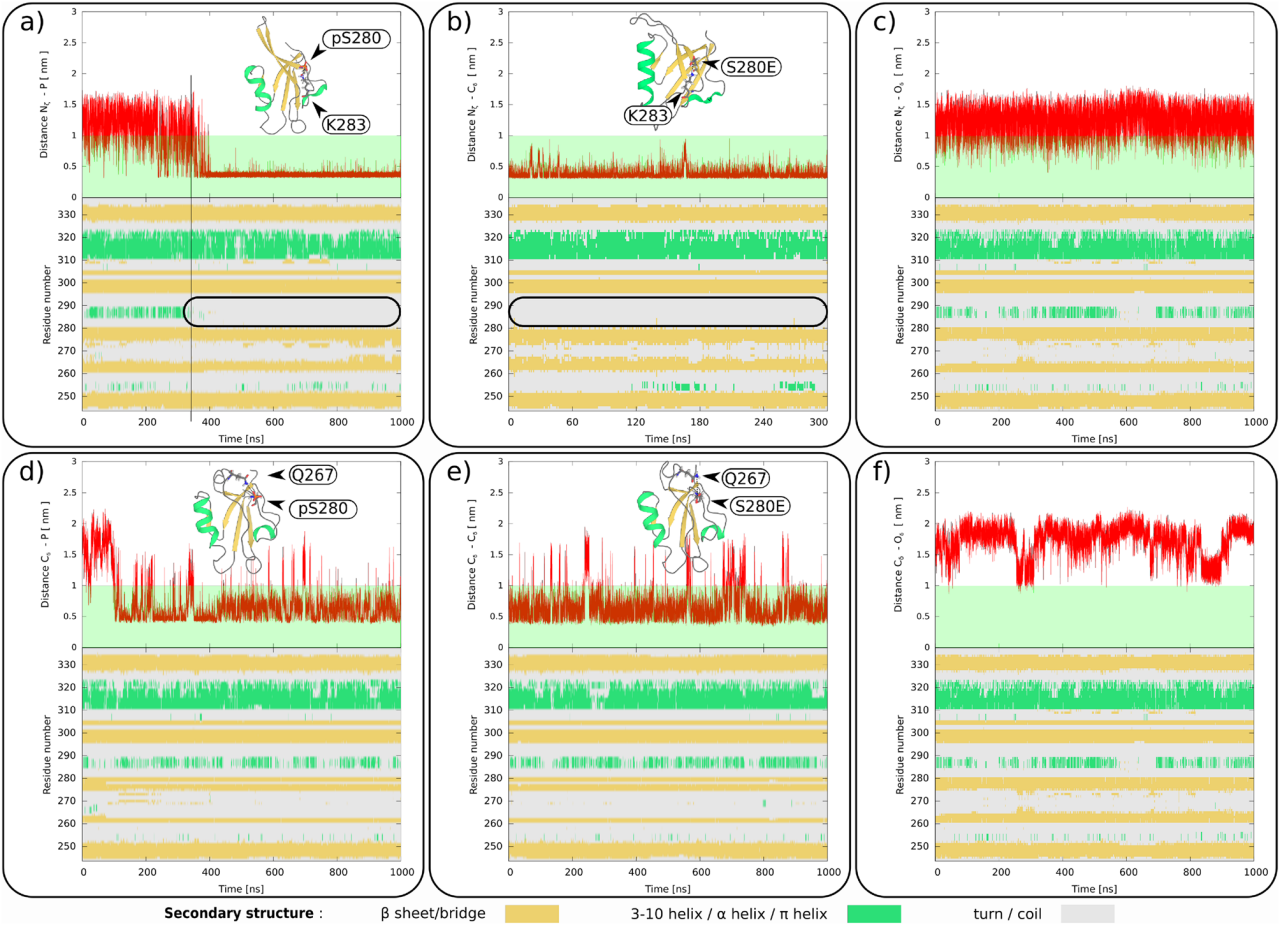
The top row (**a**–**c**) depicts the time evolution of the distances between $\hbox {N}_{\zeta }$ of K283 and (**a**) phosphate of pS280, (**b**) $\hbox {C}_{\delta }$ of S280E or (**c**) $\hbox {O}_{\delta }$ of S280 in pS280, S280E and wt systems respectively. The bottom row (**d**–**f**) depict the time evolution of the distances between $\hbox {C}_{\delta }$ of Q267 and (**d**) phosphate of pS280, (**e**) $\hbox {C}_{\delta }$ of S280E or (**f**) $\hbox {O}_{\delta }$ of S280 in pS280, S280E and wt systems respectively. Snapshot of the PDZ with highlighted interacting residues is displayed in the inlet. In the bottom of each panel is the time evolution of the PDZ secondary structure (color coding: below the picture).

### Phosphorylation of Serine 263

First of all, we examined the phosphorylation of S263. S263 resides on strand $\beta 2$ of the PDZ fold that lines one side of the ligand binding groove. In the simulations, the phosphorylation of this residue results in an electrostatic interaction across the binding groove with R320 on the $\alpha 2$ helix, the segment that lines the other side of the groove (see Fig. 3a). This interaction was accompanied by the unfolding of the C-terminal region of the $\alpha 2$ helix. Subsequently, a series of unfolding/refolding events appeared in the simulations, that seemed to be related to the formation/disruption of the interaction across the binding groove.

The interaction of the S263E mimetic with R320 was weaker compared to the phosphoserine, and was only transient in our simulations (see Fig. 3b). Although we observed a partial unfolding of the $\alpha 2$ helix and the occasional formation of an S263E–R320 interaction, the interaction was unstable in the mimetic. To test whether the discrepancy could be due to the kinetic barrier imposed by helix unfolding, we also started an independent simulation from the last conformation of the pS263 in which the $\alpha 2$ helix was already unfolded. This conformation stabilized the S263E–R320 interaction, however the stability remained lower compared to the phospho variant (see Fig. S4). Thus, the mimetic behavior was between that of phosphoserine and that of the wt, in which neither the unfolding nor the interaction across the binding groove occurred (see Figs. 3b,c or  S3).

**Figure 5:**
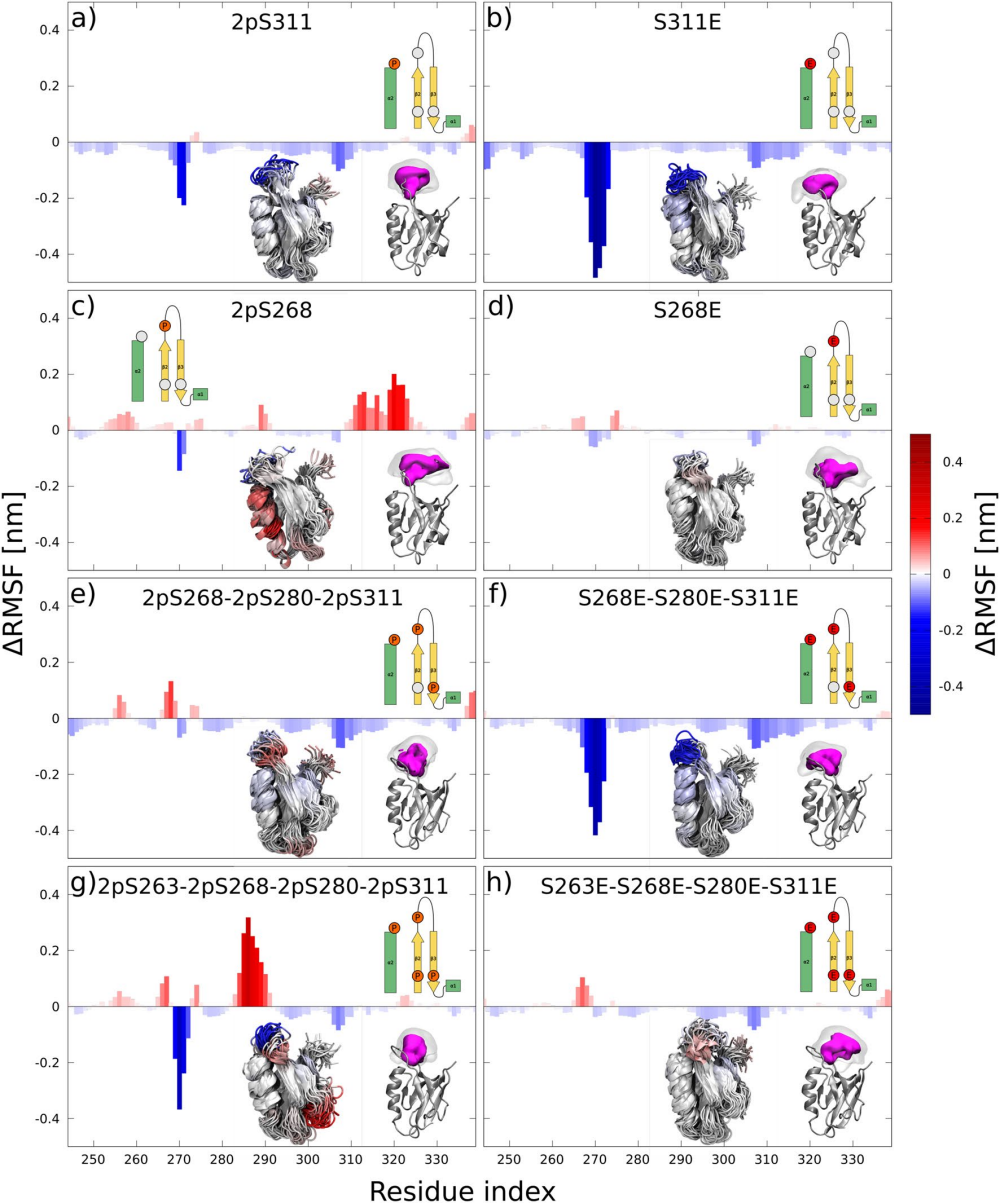
Phospho/mimetics discrepancy in multi-phosphorylated PDZ. Changes in PDZ fluctuations ($\Delta$RMSF) caused by phospho/mimetic variants. Positive values (red) and negative values (blue) corresponds to an increase and decrease in RMSF compared to PDZ wt, respectively. (**a**,**c**,**e**,**g**) and (**b**,**d**,**f**,**h**) corresponds to phospho and mimetic variants, respectively. Schematic representation of the system according to Fig. 1 is shown in the top part of each figure. A bundle of simulation snapshots colored according to RMSF change and a picture of the volume occupied by the loop $\beta 2$–$\beta 3$ (for color coding see Fig. 2) are displayed in the bottom left and right, respectively.

### Phosphorylation of serine 280

Secondly, we investigated the phosphorylation and mimetics of S280. S280 is situated on the $\beta 3$ strand adjacent to the S236 situated on the $\beta 2$ strand that lines one side of the binding groove. In simulations of pS280/S280E we observed an unfolding of the $\alpha 1$ helix and change in the position of the long $\beta 2$–$\beta 3$ loop. The unfolding or loop repositioning were induced by the interaction with either K283 situated in the short $\beta 3$–$\alpha 1$ loop or Q267 situated in the long $\beta 2$–$\beta 3$ loop. Only one of the two interactions occurred at the time.

The $\alpha 1$ helix is situated just after the $\beta 3$ strand, and its N-terminal residues are in direct contact with the carboxyl binding loop in the binding pocket. The observed unfolding of the $\alpha 1$ helix was preceded by breaking the salt bridge D290-R292 that stabilizes the short loop just after the $\alpha 1$ helix (see Fig. S5). The unfolding was especially fast in pS280, where the interaction with K283 was strong and once formed, it remained bound until the end of the 1-$\upmu \hbox {s}$-long simulation (see Fig. 4a). In contrast to pS280, the S280E formed a much less stable interaction with K283. Thus, the unfolding of the $\alpha 1$ helix was not observed in either of two independent simulations (see a) sim 1 and sim 2 in Fig. S6). However, the mimetic stabilized the unfolded state when the simulation was started from the conformation with an unfolded $\alpha 1$ helix (final snapshot of the pS280 simulation where the helix was unfolded) (see Fig. 4a,b) sim 3 in Fig. S6). This $\alpha 1$ helix folding/unfolding was also commonly observed in the wt at $\approx$ 30–80 ns timescales, but the helix always refolded back and renewed the salt bridge D290-R292 when pS280/S280E was not present (see Fig. 4c).

The long $\beta 2$–$\beta 3$ loop terminates the binding groove on the site opposite the carboxyl binding loop, and its repositioning via interaction with Q267 was observed in both the phosphorylated and mimetic simulations. The interaction of pS280/S280E and Q267 caused the loop to partially fold back on the PDZ and stabilize its structure (see Fig. 4d,e). This loop stabilization was missing when the pS280/S280E interacted with K283, whose interaction was more stable than the interaction with Q267, or in the wt (see Fig. 4f).

### Phosphorylation of serines 268 and 311

The last set of phosphorylations sites that we studied were S311 and S268. S268 and S311 are both situated at the end of the binding groove opposite the carboxyl binding loop on the $\beta 2$ strand and $\alpha 2$ helix, respectively. Thus, both sites are in contact with flexible $\beta 2$–$\beta 3$ loop whose dynamics together with the $\alpha 2$ helix were altered in our simulations by S268 and S311 phosphorylation or mimetic mutation. The pS311 and S311E increased the long $\beta 2$–$\beta 3$ loop’s rigidity and stabilized its conformation at the midpoint and at the $\alpha 2$ helix, respectively. In contrast, pS268 and S268E caused destabilization and no effects on the PDZ, respectively (see Fig. 5a–d).

The effects of pS268/S268E were smaller than those caused by the modifications of S311, and the phosphorylated variant differed from its mimetic counterpart. While pS268 resulted in an increased flexibility of the long $\alpha 2$ helix (due to the partial helix unfolding and reorientation, see Fig. S7a-b), its mimetic variant caused no substantial changes with respect to the wt. The helix unfolding and change in its orientation was initiated by a disruption of hydrogen bonds between the $\beta 2$–$\beta 3$ loop and the $\alpha 2$ helix, which enabled the helix to slide along the PDZ core and partially unfold (see Fig. S7c).

### Phospho/mimetics discrepancy in multi-phosphorylated PDZ

**Figure 6:**
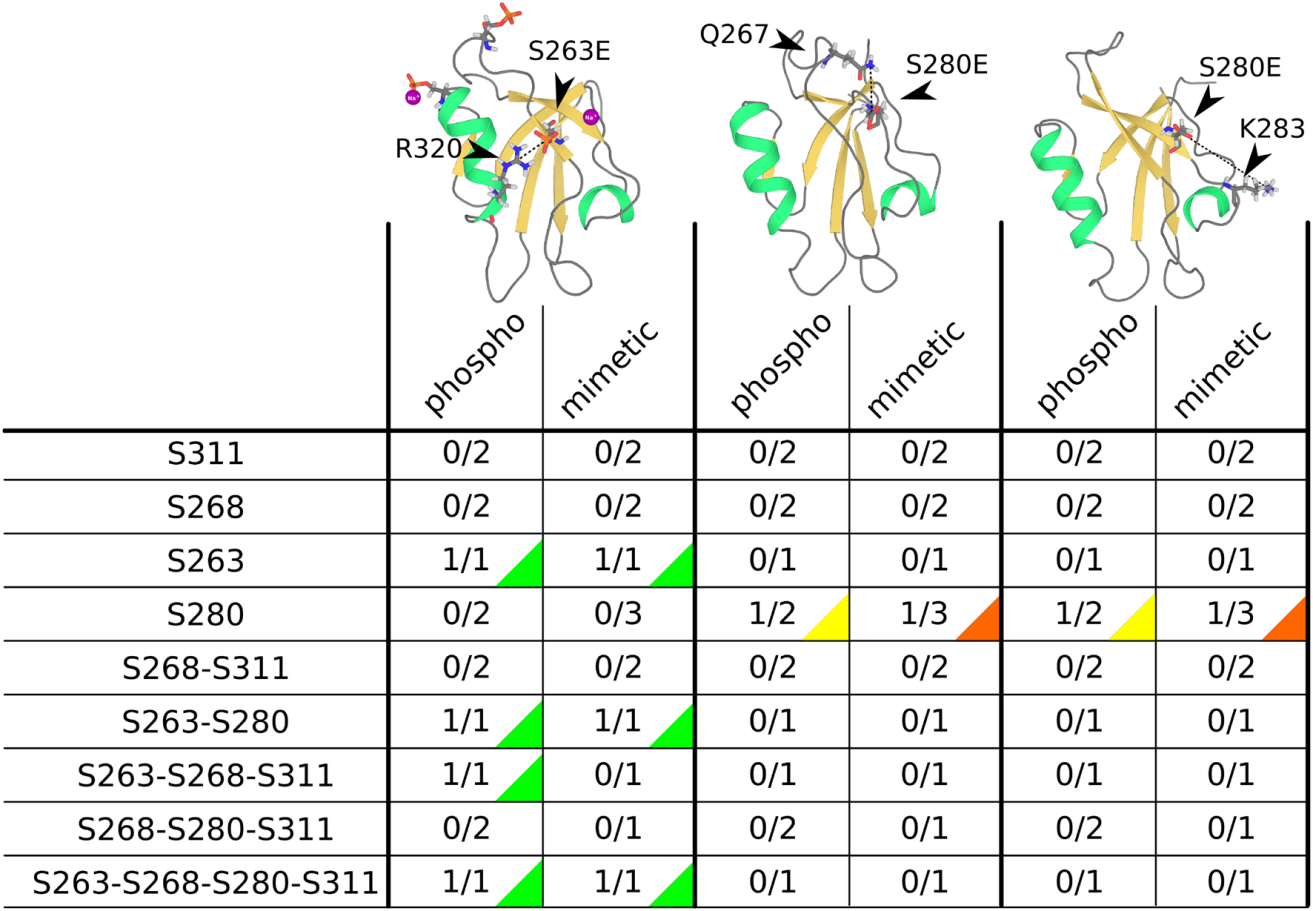
Occurrence of the three specific interactions (with R320, Q267, or K283) in simulations of different phospho/mimetic variants of PDZ. Each line corresponds to one phospho/mimetic variant. The columns represent specific interactions: interaction across the binding grove with R320, interaction with Q267 in the long $\beta 2$–$\beta 2$ loop, and interaction with K283 in the short $\alpha 1$–$\beta 2$ loop. For each column, the phospho and mimetic variant are displayed on the left and the right side, respectively. The displayed rations correspond to the number of simulations with the interaction versus the total number of simulations. Each interaction was counted only when it lasted for at least of 20 ns and was present in more than 25% of the simulation time.

As the next step we performed MD simulation of individual combinations of four phosphorylated sites. We found that the discrepancy between the phospho and mimetic variants increased with the number of modified sites and structural effects were not additive, thus could not be derived from single phosphorylations/mimetics (see Figs. 6 and S8).

In simulations, phosphorylations/mimetics that were situated in spatial proximity to each other interacted via ion-mediated interactions that reduced ability of both phosphate/carboxyl groups to interact with other residues. For example, the interaction between pS263 and pS280 or S263E and S280E was mediated by two stable $\hbox {Na}^+$ ions or one diffuse (weakly binding) $\hbox {Na}^+$ ion in the mimetic or phospho variant, respectively (see Fig. S9a). Another case was the triple phospho mutant (pS268–pS280–pS311), where the loop stabilization was mediated via two structurally stable and two diffuse (weakly binding) $\hbox {Na}^+$ ions (see Fig. S9b). Non-additivity could be also seen in pS268–pS280–pS311, where the phosphorylation of loop residues (S268 and S311) diminished the pS280 interaction with Q267 (see Fig. 6).

An example of the discrepancy between phospho and mimetics was clear in the pS263–pS268–pS311 simulation, where the pS263–R320 interaction was not affected by phosphorylation in the loop, but the interaction was diminished in the mimetic (S263E–S268E–S311E) (see Fig. 6). Also, in pS268–pS280–pS311 and S268E-S280E-S311E, we found no changes to the $\beta 2$–$\beta 3$ loop dynamics and its stabilization, respectively (Fig. 5e,f). The loop stabilization was due to the hydrogen bond network between the loop and the N-terminal region of the $\alpha 2$ helix that was only observed in the mimetic (see Fig. S10). Even more profound differences between the phospho and mimetic variant were found in a simulations with all four sites (S263, S268, S280, and S311) modified. The phospho variant exhibited substantially increased flexibility in the $\alpha 1$ helix due to its unfolding (see Fig. 5g,h), similar to the single pS280 (see Fig. S5), which was not observed in the mimetic. Interestingly, the pS280/S280E interaction with K283 in the $\alpha 1$–$\beta 2$ loop was only observed when no other sites were phosphorylated or mutated.

### NMR titration

**Figure 7:**
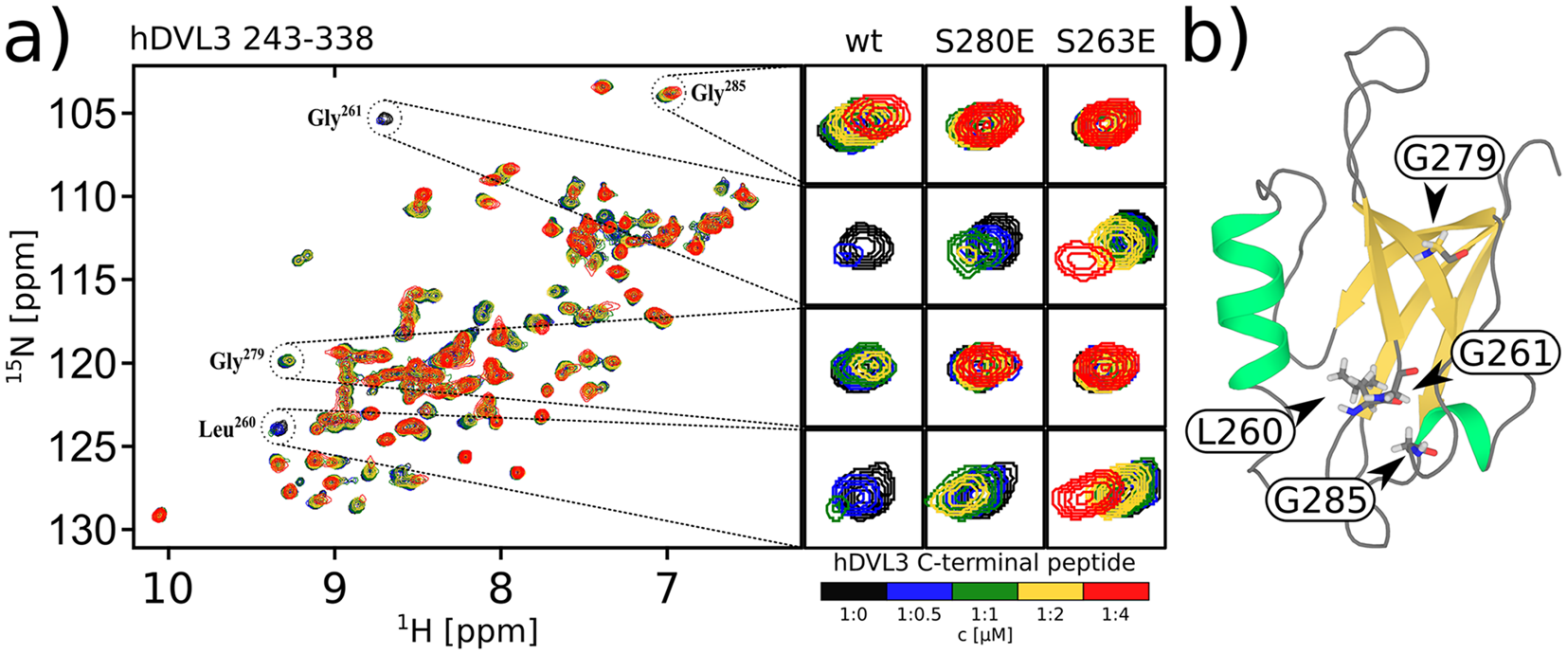
The NMR titration. (**a**) Overlay of $^{1}\hbox {H}$–$^{15}\hbox {N}$ HSQC spectra from NMR titration of the PDZ domain (243–338) from human DVL3 by C-terminal peptide (698–716) (see “Materials and methods” section). Spectra with increasing concentration of the C-terminal peptide are colored from black (no peptide) to red (four-time higher concentration of peptide with respect to PDZ). Peaks that report PDZ-peptide interaction are highlighted with circles. Changes in peak intensity for each reporter residues in mutants S280E and S263E are emphasized on the side. (**b**) Position of reporter residues in the PDZ domain.

MD analysis suggested that the phosphorylation/phosphomimetic of S263 and S280 could interfere with ligand binding. We thus decided to test the predictions experimentally. The effect of S263E and S280E mutants on the DVL3 PDZ (aa243–338) affinity towards a peptide from its own C-terminus (aa 698-716) was tested by NMR titration experiments (see Fig. 7 and Fig. S11). The monomeric state of the PDZ domain during the NMR titration was verified by analytical centrifugation measurements (see Fig. S13). Centrifugation was performed at 25 and $100~\upmu \hbox {M}$ concentrations of PDZ, where the highest concentration is similar to our NMR experiments. To asses PDZ binding, we used reporter residues that are resolved in the HSQC spectra. Two of them are situated in the carboxyl binding loop (L260 and G261) that participates in hydrogen bonding with the terminal carboxyl group of the ligands^11^, and two other residues that lie adjacent to the binding groove and are perturbed by peptide binding (G279 and G285). The chemical shift perturbations of all residues in all PDZ mutants and wt are shown in supplementary information (see Fig. S12). For both loop residues, we observed a rapid decrease in signal intensity with increasing peptide concentration for the wt PDZ domain. Similarly, in the S280E mutant, the peak intensities decreased with increasing peptide concentration, but broadened beyond detection at higher peptide concentrations than the wt. The corresponding peaks of the S263E mutant, however, experienced hardly any broadening. A similar trend was observed for the peaks on the periphery of the binding groove: a fast to intermediate exchange for the wt PDZ domain but no chemical shift perturbations for either phosphomimetic variant. Therefore, the peptide affinity for PDZ is clearly attenuated by the phosphomimetic mutants and could be ordered as wt>S280E>S263E, with S263E having a very weak affinity towards the peptide. This data confirms the conclusions drawn by the MD simulations, and suggests that S263 phosphorylation can regulate the PDZ binding to target proteins.

### Rescue analysis in DVL triple knockout cells

**Figure 8:**
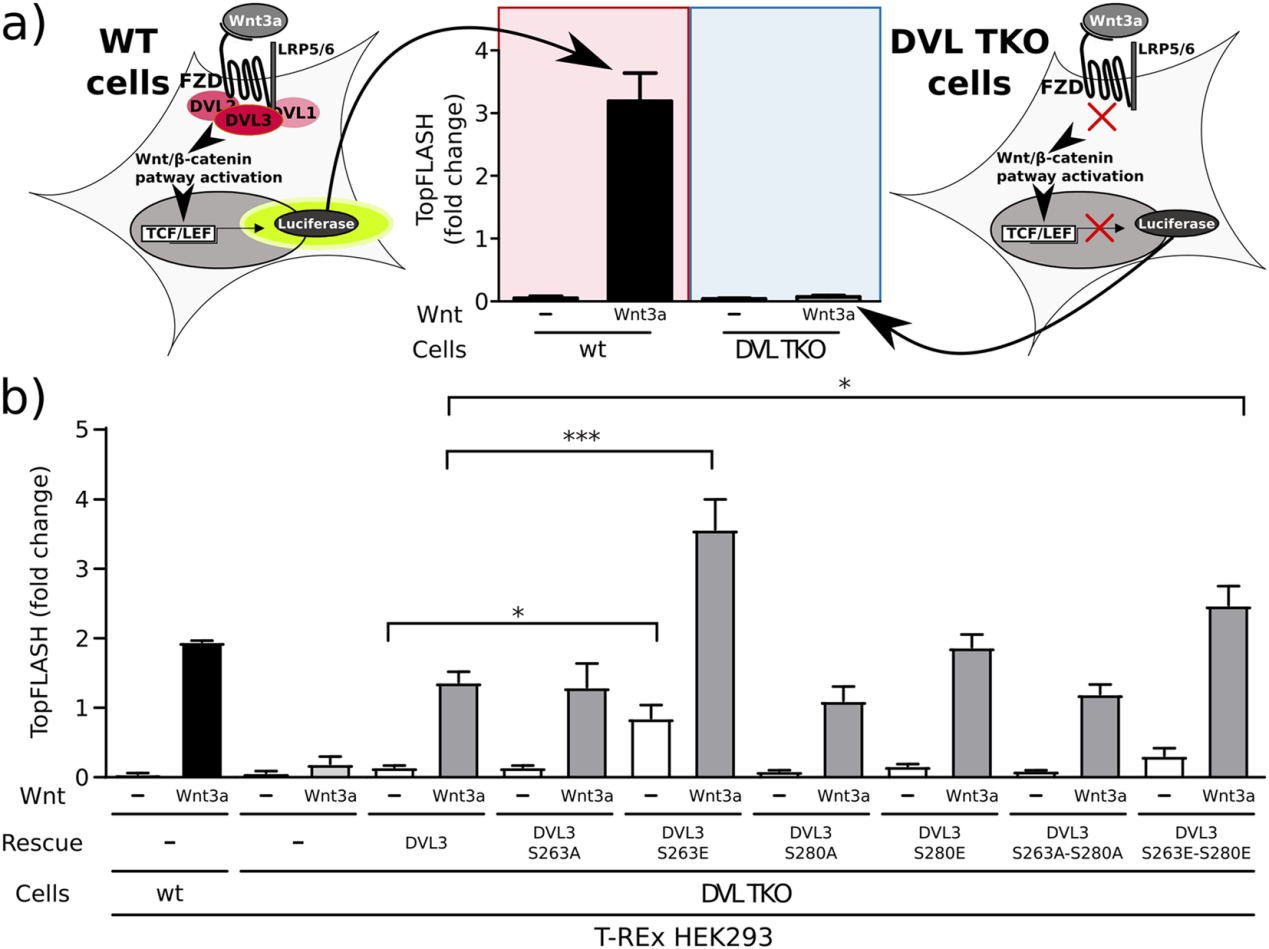
Rescue analysis in DVL triple knockout cells. (**a**) The illustration of the DVL3 rescue TopFlash assay where luciferase activity is measured after addition of Wnt3a ligand. If active DVL molecules are present in the cell a change of luminescence is measured. (**b**) The luciferase activity in different DVL3 mutants. All phosphomimicking (S–E) or phospho-preventing (S–A), used as a negative control, DVL3 mutants were able to rescue transcriptional response in DVL TKO cells induced by recombinant Wnt3a. However, DVL3 S263E and DVL3 S263E/S280E mutants were hyperactive upon Wnt-3a stimulation. Interestingly, DVL3 S263E behaved as constitutively active and activated TopFlash response even in the absence of Wnt-3a as analyzed by TopFlash assay, $\hbox {n}=3$ with SEM indicated by error bars. The statistical differences were analyzed by paired Student’s t-test (*${p}<0.05$, **${p}<0.01$).

The effect of the structurally most different PDZ variants—S263E, S280E and S263E–S280E—on the activation of the Wnt/$\beta$-catenin signaling pathway was tested in cell assays. We decided to use rescue assays in triple *DVL1/DVL2/DVL3* knockout HEK293 T-Rex (DVL TKO T-REx) cells^35^. In this assay, Wnt3a signal transduction—analyzed by Dual-Luciferase TopFlash reporter gene assay (for schematics see Fig. 8a)—is disrupted and can be rescued by the re-expression of the wild type or mutated DVL variant. This assay eliminates some of the overexpression artefacts and interference from the endogenous wild-type DVL present in cells. It is known that the PDZ domain binds the DVL3 C-terminus^36^. Such a closed conformation of DVL3 leads to autoinhibition that is released by activation by the Wnt pathway^32^. Interestingly, the DVL3-S263E mutant caused the Wnt pathway to be active even in the absence of Wnt3a activation, and appeared to be also hyperactive in comparison to the wt upon pathway activation by Wnt3a, which suggests that it is in the open conformation. DVL3-S280E behaved like the wild type. In the double mutant (DVL3-S263E–S280E) we did observe a slight activation even in the absence of Wnt3a, and similarly to the S263E mutant, observed an increased activation level after the addition of Wnt3a with respect to the wt. To test if the observed effects were specific to mimetic we prepared alanine mutants as controls. All controls were able to rescue cells and had response similar to wt DVL3 (see Fig. 8b). Western blot analysis was performed to confirm comparable expression levels of all transfected constructs (see Fig. S14). These data are in agreement with the NMR experiments and simulations, and suggest that indeed S263E-(S280E) DVL3 is deficient in binding its own C-terminus, stays in the open conformation, and behaves as a constitutively active form.

## Discussion

The PDZ domain is a protein-protein recognition motif that is crucial for many biological processes^37^. We investigated the effects of phosphorylation on the PDZ domain in the third isoform of human Dishevelled protein (hDVL3), which is a crucial component^29^ of an ancient and evolutionarily conserved Wnt signaling pathway. Phosphorylation plays a crucial role in Wnt signaling and in DVL regulation, where upon activation DVL gets heavily phosphorylated by a plethora of kinases. However, only recently the systematic study of DVL phosphorylation sites was performed drawing a connection between particular kinases and their respective phosphorylation sites^31^. Here we studied a subset of these residues located in the Dishevelled PDZ domain. We characterized the effects of single and multiple phosphorylations and corresponding mimetic mutants. An uncommon approach in the field of PDZ domains, where most of the current phosphorylation studies focus on PDZ ligands^38–41^ and effects of direct PDZ phosphorylation remains scarce^34^.

In simulations, we studied the four phosphorylations sites S263, S268, S280, and S311, together with their phospho mimetics. The PDZ phosphorylations appeared to have three major effects on the PDZ structure: an electrostatic interaction that bridged the canonical binding groove, a change in the conformation of the $\beta 3$–$\alpha 1$ loop, and changes in the PDZ flexibility, particularly of the $\beta 2$–$\beta 3$ loop.

We observed an interaction across the binding groove between R320 and pS263/S263E in our simulations. This interaction could obstructs access to the PDZ binding site and suggested a reduced affinity to the ligand. Indeed, a reduction in affinity was obtained between PDZ S263E and the C-terminus ligand in the NMR titration experiment. Despite the agreement with our simulations, our NMR results do not demonstrate that the ligand binds to the binding groove of PDZ. Nevertheless, this is expected as the C-terminus of DVL was previously shown to bind to the PDZ binding groove in DVL1 and the ligand C-terminus differed from ours only by a single isoleucine to valine mutation^36^. Moreover, our DVL3-S263E mutant exhibit a distinctive phenotype, in vitro and behaved as a constitutively active form. Such activation is in line with the reported ‘open/closed’ states of DVL^36^ and the recently published study of DVL, where phosphorylation reduced the binding of the C-terminus to PDZ, resulting in DVL activation in vivo^32^. Furthermore, a similar bridge across the binding groove was previously described in the experimental study of INAD PDZ5, where a disulfide bridge was formed across the binding groove that obstructed access to the binding site^12^. In INAD PDZ5 crystal structure, the interaction was accompanied by a partial unfolding of the $\alpha 2$ helix, which was also observed in our simulations.

Our simulations suggest that modifications of S280 had more subtle effects on PDZ structure and ligand binding than S263. We found that both S280E and pS280 participate in the two types of interactions with either K283 from the $\beta 3$–$\alpha 1$ loop or Q267 from the $\beta 2$–$\beta 3$ loop. The interaction with K283 was relatively strong, long-lasting, and related to the pronounced unfolding of the $\alpha 1$ helix. In contrast, the interaction with Q267 was less stable, with fast binding/unbinding dynamics, and resulted in loop stabilization. Both of these interactions could affect the ligand binding indirectly. Changes in the $\alpha 1$ helix were experimentally reported to allosterically modify the binding site^42–45^ and together with the $\beta 3$–$\alpha 1$ loop can participate in signal transduction pathways^46,47^. Furthermore, unfolding/rearrangement of $\alpha 1$ helix region might be important for the interaction of PDZ with other binding partners similarly as in Par-6 PDZ, where a partial unfolding was experimentally observed^48^. The $\beta 2$–$\beta 3$ loop stabilization could also affect the ligand binding because it is known to be a typical secondary binding site^6,19,49–53^, which is usually stabilized upon ligand binding^3,54,55^. In agreement with our simulations, the NMR data demonstrated slightly reduced ligand binding in the S280 mutant compared to the wt. Accordingly, S280E fully rescues cell response to the Wnt/b-catenin signalling with a slightly higher response to the activator Wnt3a compared to the wt, which could be caused by the reduced affinity towards the C-terminal ligand. Note that our simulations suggest this effect to be stronger for phosphorylated PDZ in vitro.

In all other simulations, the effects on PDZ were smaller and could not be deduced from the effects of individual modifications of S263 and S280. Thus in general, our simulations suggest that phosphorylations other than S263 and S280 seem to have a modulating effect on the PDZ structure and could fine-tune its interactions. Interestingly, a similar effect of multiple phosphorylations on protein structure was observed in a recent simulation study where one phosphorylation had a strong effect while the other only modulated the effect further^56^.

In simulations, we observed that different phospho/mimetic combinations had non-additive effects on both PDZ’s structure and its flexibility which was shown to be important for PDZ recognition^57^. In general, by increasing the number of phospho/mimetics in the PDZ, the interactions observed in single phospho/mimetic variants were either not affected or weakened, when further modifications were far from or close to the initial ones. For example, when both $\beta$-sheet residues S263 and S280 were modified simultaneously, their interaction capacity with other residues was reduced compared to systems with a single modification because of ion-mediated interaction between the residues. This reduction is in line with observations in the Wnt3a-dependent reporter assay, where the S263E–S280E double mutant had reduced activity compared to the S263E single mutant.

Our simulations shown an interaction mediated by positively charged sodium ions between two close negatively charged side-chains. Such ion-mediated interactions were occasionally found in mimetic and more often in phosphorylated systems due to the higher negative charge density of phosphate. The interactions of pS263/S263E with pS280/S280E and of pS268/S268E with pS311/S311E were the most significant. Note that the observed ion-mediated interaction might be too strong for sodium ions in the current force-field^58^, but similar or even stronger interactions are expected to occur in the presence of multivalent ions such as $\hbox {Ca}^{2+}$, which strongly interact with phosphate^59^. Moreover, the ion-mediated interactions are likely to be environment- and salt-dependent and could be used as an additional regulation mechanism. For example, in the non-canonical Wnt/$\hbox {Ca}^{2+}$ signaling, $\hbox {Ca}^{2+}$ ions serve as second messengers, and thus such interaction could serve as feedback regulation mechanism^60,61^.

In all simulations, the mimetics seemed to have a relatively stabilizing effect on the PDZ domain compared to phosphorylations, which in certain cases induced local PDZ disruption. The stronger effect of phosphoserine compared to glutamic acid could originate from its stronger electrostatic interaction, in particular when the phosphate is doubly deprotonated (charge − 2e). Therefore, the newly formed interactions between a phosphate group and arginine^62,63^ or lysine^63^, e.g. R320 with pS263 or K283 with pS280, were stronger than the mimetics.

In general, our simulations suggest that single site phosphorylations or mimetics have similar effects with the exception of S268. In such a case, we observed a large-scale rearrangement of the long $\alpha 2$ helix occurring only in a phosphorylated system.

In systems with two or more modified phosphorylation sites, the differences between phosphorylated and mimetic variants increased with the increasing number of modified sites. The highest discrepancy was observed in the simulation with all four sites modified (S263–S268–S280–S311), where the mimetic behaved very similar to wt, while the phosphorylated version differed from wt in both the $\beta 2$–$\beta 3$ loop and the short $\alpha 1$ helix.

To conclude, we studied phosphorylation-induced structural changes in the PDZ domain from Dishevelled protein, a key component of Wnt signaling pathways. We focused particularly on the third isoform of human DVL protein modified at four recently discovered phosphorylation sites in PDZ in vitro. The phosphorylation of S263 created a salt bridge across the binding groove, which suggests the possibility of S263 being an on/off ligand binding switch regulated by NEK2 kinase. These simulation results are supported by NMR titration experiments with the S263E mimetic, which exhibited a negligible PDZ-ligand affinity compared to the wt. Moreover, the reduced PDZ-ligand affinity correlates with the constitutively activated phenotype observed for the S263E mimetic in our biological rescue assay. The phosphorylation of S280 exhibited more complex behavior, either stabilizing the $\beta 2$–$\beta 3$ loop (an extended binding site^6,19,49–53^) or inducing the unfolding of the $\alpha 1$ helix , which could allosterically influence the primary binding site^42–45^. Such structural modifications of PDZ are in agreement with the reduced PDZ-ligand affinity found for the S280E mimetic in NMR. The simultaneous phosphorylation of PDZ at different sites increased the complexity and non-additivity of observed effects, and amplified differences between mimetics and phosphorylation variants. The most apparent non-additive effect was found in PDZ with pS263 and pS280, where phosphates did not behave independently because of salt/pH dependent phosphate-cation-phosphate interaction. The obtained understanding of the phosphorylation-induced changes to PDZ at atomistic resolution could be useful for insights into other PDZ domains and could bring us closer to understanding DVL and Wnt regulation.

## Materials and methods

### Simulations

The wild-type PDZ domain (aa 245-338) from hDVL3 was constructed via homology modeling using MODELLER v9.11^64,65^. As a template, we used the crystal structure of the hDVL2 PDZ (PDB code 2rey) which shares 96.28% sequence identity. Missing loops and an extra residue at the C-terminus were added via MODELLER^66^. The generated models were evaluated based on DOPE and GA341 scores, and the best structure was selected and used for the study. The homology model was stable for the length of our simulation, $1~\upmu \hbox {s}$ (see Fig. S1).

All mimetic and phosphorylated variants of the PDZ domain were prepared based on the homology model with PyMOL^67^ visualization software using the mutagenesis tool and PyMTs plugin^68^, respectively. Each phosphoryl group was considered to be fully deprotonated (charge of $-2$ e) as it should be at neutral pH if we do not account for a local environment effect^69,70^. Note that we marked all phosphoserine residues in the text with pS. Both ends of the PDZ domain were capped to reduce the effect of the protein termini. The C-terminus was capped with an acetyl group and the N-terminus with an N-methyl group. The protonation state of residues was chosen according to a neutral $\hbox {pH}=7$, with the histidine H324 being neutral. Each system was solvated with $\approx 11 \times 10^{3}$ water molecules and placed in a cubic box of size $7 \times 7 \times 7$ nm using periodic boundary conditions. NaCl ions were added a concentration of 150 mM with excess ions to neutralize the system net charge. We used the all-atom force field amber99sb-ILDN^71,72^ with the phosphoserine parameters^73^. Robert Best’s correction was employed to better describe flexible/unfolded regions such as loops by scaling van der Waals interactions between water oxygen and protein by a factor of 1.1^74^. The TIP3P water model^75^ was employed.

Once solvated, each system was minimized and equilibrated in an NVT ensemble (constant number of particles, volume, and temperature), followed by an NPT ensemble (constant number of particles, pressure, and temperature). Each equilibration was performed for 0.1 ns followed by a production simulation for at least 300 ns in the NPT ensemble. The temperature was kept at 309.15 K with a velocity-rescale thermostat^76^, using a coupling constant of 0.1 ps applied separately to the solvent and protein. The pressure was held at 1 bar via a Parrinello–Rahman barostat^77^ with a coupling time of 2 ps and compressibility of $4.5 \times 10^{-5}~{\text {bar}}^{-1}$. Simulations were carried out with a 2 fs time step with the leap-frog integrator. The direct space cut-off for both electrostatics and van der Waals interactions was set to 1 nm. Long-range electrostatic interaction was evaluated with particle-mesh Ewald summation^78,79^. Covalent bonds with hydrogen atoms were constrained with the LINCS algorithm^80^. All MD simulations were performed with the Gromacs software package 5.1.2^81,82^. To calculate the probability of interaction between two atoms, we used distance criteria of 1 nm between atoms. Unless stated otherwise, all simulations were $1~\upmu \hbox {s}$ long.

### Protein expression and purification for NMR

#### Materials

For the specific labeling of proteins, the $^{15}\hbox {NH}_{{4}}\hbox {Cl}$ and $^{13}\hbox {C}_{{6}}$ glucose was bought from Cortecnet. All the purification steps were performed on Akta pure system from GE healthcare. The chemicals used during the protein expression and purification were bought from Sigma.

#### The protocol

The DNA sequence of the PDZ domain of human DVL3 (aa 243–338) was cloned into the pETM11 vector containing an N-terminal $\hbox {His}_6$-tag separated by a tobacco etch virus (TEV) protease digestion site^83^. Site-directed mutagenesis was employed to prepare the phosphomimetic mutants. *E. coli* BL21(DE3) transformed with pETM11-PDZ (wt or mutants) was propagated in 1-l cultures of minimal media ($^{15}\hbox {NH}_4\hbox {Cl}$ 1 g $\hbox {l}^{-1}$ and $^{13}\hbox {C}_6$ glucose 2 g $\hbox {l}^{-1}$ as the sole nitrogen and carbon sources, respectively) for the uniform $^{15}\hbox {N}$- or $^{15}\hbox {N}$, $^{13}\hbox {C}$ labeling of the proteins. The bacterial cultures were grown to $\hbox {OD}_{{600}} \approx 0.8$, and protein expression was induced by the addition of 0.5 mM isopropyl $\beta$-D-1-thiogalactopyranoside at $16 ^{\circ }\hbox {C}$ overnight. Cells were lysed (lysis buffer: 25 mM Tris pH 8, 500 mM NaCl, 10 mM Imidazole and 10% glycerol) by sonication at $4^{\circ }\hbox {C}$ with 5 s pulse on and 10 sec pulse off cycle. The cell pellet was removed by centrifugation at 14,000 rpm and the lysate containing the soluble proteins was loaded on a Ni-NTA column. Bound proteins were eluted with 500 mM imidazole in a gradient manner and treated overnight with TEV protease to remove the N-terminal $\hbox {His}_6$-tag. Since all the proteins have acidic theoretical pI (4.5–4.8), the proteins were dialyzed in a low salt buffer (25 mM Tris pH 8, 50 mM NaCl) and loaded onto a Q-sepharose column. Eluted proteins were concentrated using 3 kDa centricon from vivaspin and to size exclusion chromatography column (superdex 75 10/300 GL). The untagged proteins were concentrated and transferred to the buffer for NMR or binding experiments containing 50 mM sodium-phosphate (pH 6.5) and 50 mM KCl.

### NMR spectroscopy

NMR measurements were performed in a 600 MHz Bruker Avance III spectrometer equipped with a 1H/13C/15N TCI cryogenic probehead with z-axis gradients at the CEITEC Josef Dadok National NMR Centre. Chemical shift assignments were obtained using the 4D-CHAINS technology^84^. For titration experiments, $^1\hbox {H}$–$^{15}\hbox {N}$ HSQC spectra of $100\,\upmu \hbox {M}$ PDZ wt/mutant solution were recorded with increasing amounts of C-terminal DVL peptide consisting of aa 698-716 from DVL3 with phosphorylated pS700 (stock concentration of $800~\upmu \hbox {M}$). The peptides were uncapped. The weighted $^1\hbox {H}$ and $^{15}\hbox {N}$ chemical shift differences were calculated according to the following equation

$$\begin{aligned} \Delta \delta ^{obs} = \sqrt{\left( \delta ^{1H}\right) ^2+\left( \frac{1}{6} \delta ^{15N}\right) ^2}, \end{aligned}$$

where $\Delta \delta ^{obs}$ represents the observed chemical shift difference, $\delta ^{1H}$ is the change in hydrogen chemical shift, and $\delta ^{15N}$ is the change in nitrogen chemical shift.

### Analytical ultracentrifugation

The sedimentation velocity experiments were performed in a ProteomeLab XL-I analytical ultracentrifuge (Beckman Coulter, Indianapolis, IN, USA) equipped with an An-60 Ti rotor. Measurements of sedimentation velocity were done at 60000 rpm and 20 $^\circ \hbox {C}$ in 12mm titanium double-sector centerpiece cells (Nanolytics Instruments, Potsdam, Germany). Cells were loaded with $380\,\upmu \hbox {l}$ of reference buffer (50 mM sotassium-phosphate (pH 6.5) and 50 mM KCl) and samples with hDvl3 PDZ WT domain (residues 243–338) solution. $25\,\upmu \hbox {M}$ and $100\,\upmu \hbox {M}$ protein concentrations were measured after 1.5 h equilibration in cells (see Fig.S13). Absorbance scans were collected at 280 nm in 5-min intervals with 0.003 cm spatial resolution in continuous scan mode. The SEDNTERP software^85^ was used to estimate the partial specific volume of the protein, buffer density, and buffer viscosity. The data were analyzed with the continuous c(s) distribution model in the program SEDFIT v15.01c^86^. For the regularization procedure, a confidence level of 0.68 was used. The plots of c(s) distributions were created in GUSSI 1.3.1^87^.

### Triple-knockout experiments

#### Materials

Cell cultures: Dulbecco’s modified Eagle’s medium, Gibco, Life Technologies, REF41966-029, fetal bovine serum (Gibco, Life Technologies, 10270-106), penicilin/streptomycin (Biosera, XC-A4122/100), L-glutamin (Life Technologies, 25030024), inhibitor LGK974 (Stem RD, 974-02), Wnt3a recombinant protein (R&D Systems, 1324-WN, 645-WN), recombinant human R-spondin 1 (PeproTech, 120-38). TopFLASh assay: Dual-Luciferase^®^ Reporter Assay System (Promega, E1960). Antibodies: FLAG M2 (F1804) from Sigma, $\beta$-actin (Cell Signalling, 4970). Mutagenesis: QuikChange II XL Site-Directed Mutagenesis Kit (Agilent).

#### Cell culture and transfection

HEK293 T-REx cells [human embryonic kidney (wt T-REx cells)], *DVL1/DVL2/DVL3* KO HEK293 T-Rex (DVL TKO T-REx described in^35^) were cultured in Dulbecco’s modified Eagle’s medium (DMEM; catalogue number 41966-029; Gibco, Life Technologies) with the addition of 10% fetal bovine serum (FBS; catalogue number 10270-106; Gibco, Life Technologies), 1% penicillin-streptomycin (catalogue number XC-A4122/100; Biosera), and 1% L-glutamine (catalogue number 25030024; Life Technologies).

#### Plasmids and site directed mutagenesis

The plasmids used were described previously: pCDNA3.1-Flag-hDVL3^88^, pCDNA3.1-Flag-hDVL3 S280E^89^, and Super8X TOP Flash^90^. The site-directed mutagenesis of pCDNA3.1-Flag-hDVL3 was performed with a QuikChange II XL Site-Directed Mutagenesis Kit (Agilent) according to the manufacturer’s instructions. Mutagenesis primers used: pCDNA3.1-Flag-hDVL3 S263E: FW

TATAACTTCTTGGGCATCGAGATTGTGGACCAAAGCAAC, RV:

GTTGCTTTGGTCCACAATCTCGATGCCCAAGAAGTTATA. All plasmids were verified by Sanger sequencing.

#### Dual-luciferase assay, Western blot

For transfection, cells were seeded at 150,000 cells/well in a 24-well plate. On the next day, the cells were transfected using polyethylenimine (PEI) at a concentration of $1\,\upmu \hbox {g/ml}$ and pH 7.4 and a PEI ratio of $6\,\upmu \hbox {l}$ of PEI/$1\,\upmu \hbox {g}$ DNA. The mixture of transfected plasmids and PEI was diluted separately in plain DMEM (DMEM without FBS, L-glutamine, and antibiotics), incubated at room temperature for 30 min, afterwards plasmid DNA and PEI were mixed. The total transfection mix volume was $50\,\upmu \hbox {l}$ per well. Samples were vortexed, centrifuged, and incubated for 30 min at room temperature and then added to the cells. After 6 h, the medium containing the transfection mix was removed and replaced with complete DMEM. In all experiments, cells were treated with $1\,\upmu \hbox {M}$ Porcupine inhibitor LGK974 (catalogue number 974-02; Stem RD) to diminish the autocrine secretion of all Wnt ligands. The concentrations of pRLtkLuc plasmid and Super8X TopFlash were $0.1\,\upmu \hbox {g}$ DNA per well, the concentration of indicated Dvl plasmid was 10 ng per well in a 24-well plate and equalized with pcDNA 3.1 to $0.4\,\upmu \hbox {g}$ per well. For the stimulation of the cells, 80 ng of recombinant human Wnt3a (Wnt3a) (R&D Systems, 5036-WN) was used. All cells were treated with recombinant human R-SPONDIN1 (catalogue number 120-38; PeproTech) at a concentration of 250 ng/ml for at least 14 h. Cells were then analysed with the Dual-Luciferase reporter assay system (catalogue number E1960; Promega) according to the manufacturer’s instructions. Luminescence was measured with a Hidex Bioscan Plate Chameleon luminometer. Data were analysed with Microsoft Excel and GraphPad Prism software. Immunoblotting and samples preparation was performed as previously described^91^. The antibodies used were Actin (4970) from Cell Signaling Technologies and FLAG M2 (F1804) from Sigma.

## Supplementary Information

Supplementary Figures.
