# Supplementary material for: Phosphorylation-induced changes in the PDZ domain of Dishevelled 3
Source: Sci Rep. 2021 Jan 15;11:1484. doi: 10.1038/s41598-020-79398-5 (PMC7810883; doi:10.1038/s41598-020-79398-5)
Supplement: Supplementary file 1 — Supplementary Figures. [file 41598_2020_79398_MOESM1_ESM.pdf]

Supporting Information

Phosphorylation-induced changes in the PDZ domain of Dishevelled 3

Miroslav Jurásek, Jitender Kumar, Petra Paclíková, Alka Kumari, Konstantinos Tripsianes, Vítězslav Bryja, and Robert Vácha

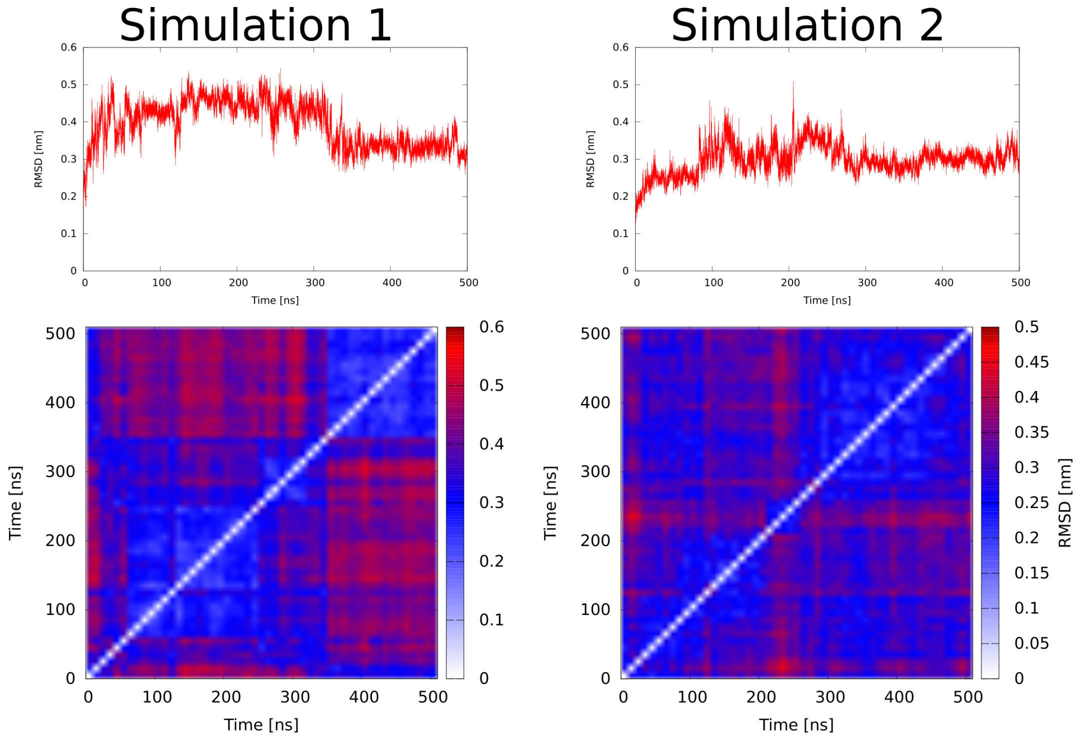

Figure S1. The RMSD time dependence of two best homology models. RMSD is calculated during two 500 ns long simulations. Simulations were started from two best structures derived from the modeling (the difference were mainly in the long loop conformation). In the bottom, RMSD matrix where RMSD between all simulation frames is calculated.

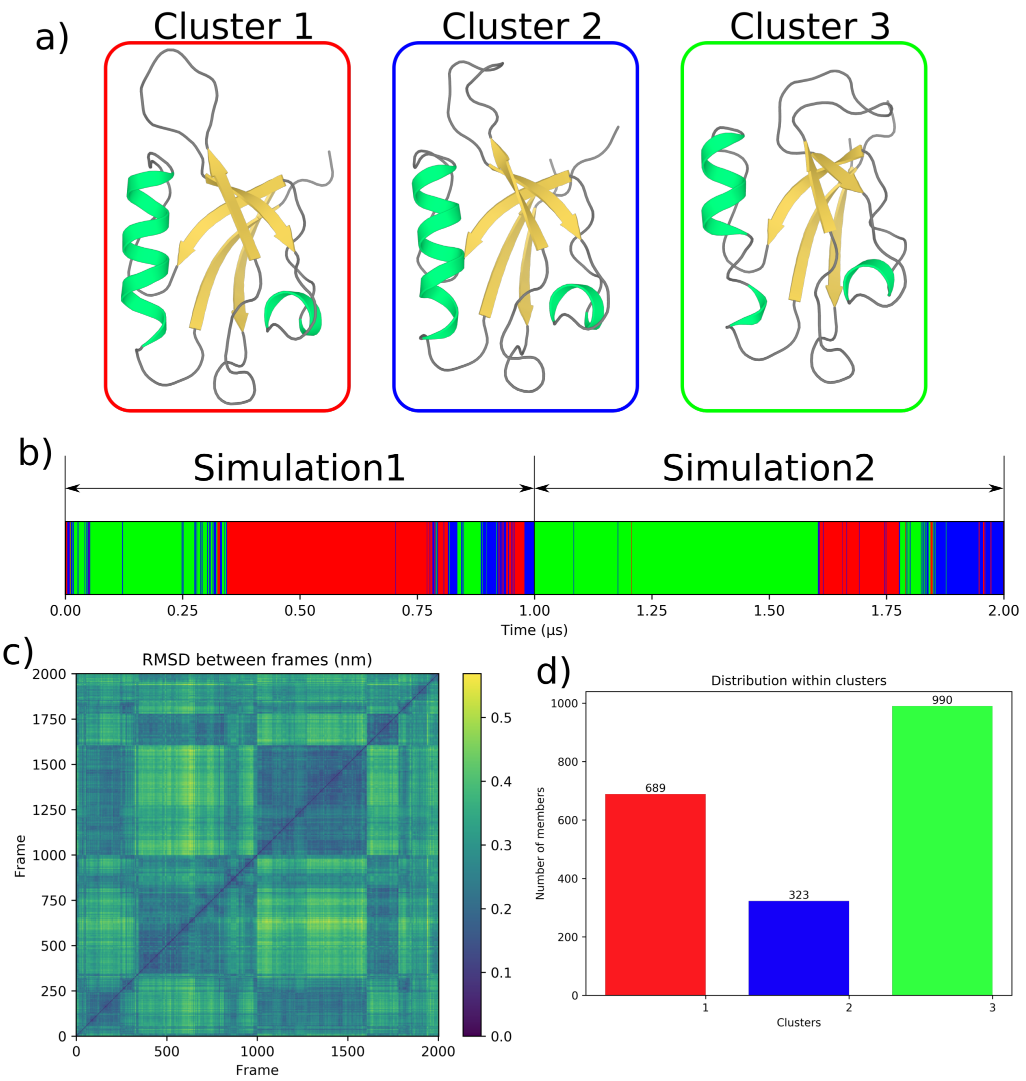

Figure S2. Clusters of PDZ wt from two 1 μs long free simulations. a) Three observed cluster structures, rectangle color is used in the whole figure to identify each cluster. Clusters differ mostly in the loop conformation especially cluster 3 with its loop conformation reaching towards the PDZ C and N termini. b) Time evolution of cluster occurrence, where the first half and the second half correspond to two separate 1 μs simulations. c) RMSD matrix between snapshots from two PDZ wt simulations. d) An abundance of each cluster. Simulation clustering and analysis was done with program from ref: [Tubiana, T., Carvaillo, J.-C., Boulard, Y. Bressanelli, S. TTClust: A versatile molecular simulation trajectory clustering program with graphical summaries.J. Chem. Inf. Model.58, 2178–2182 (2018)].

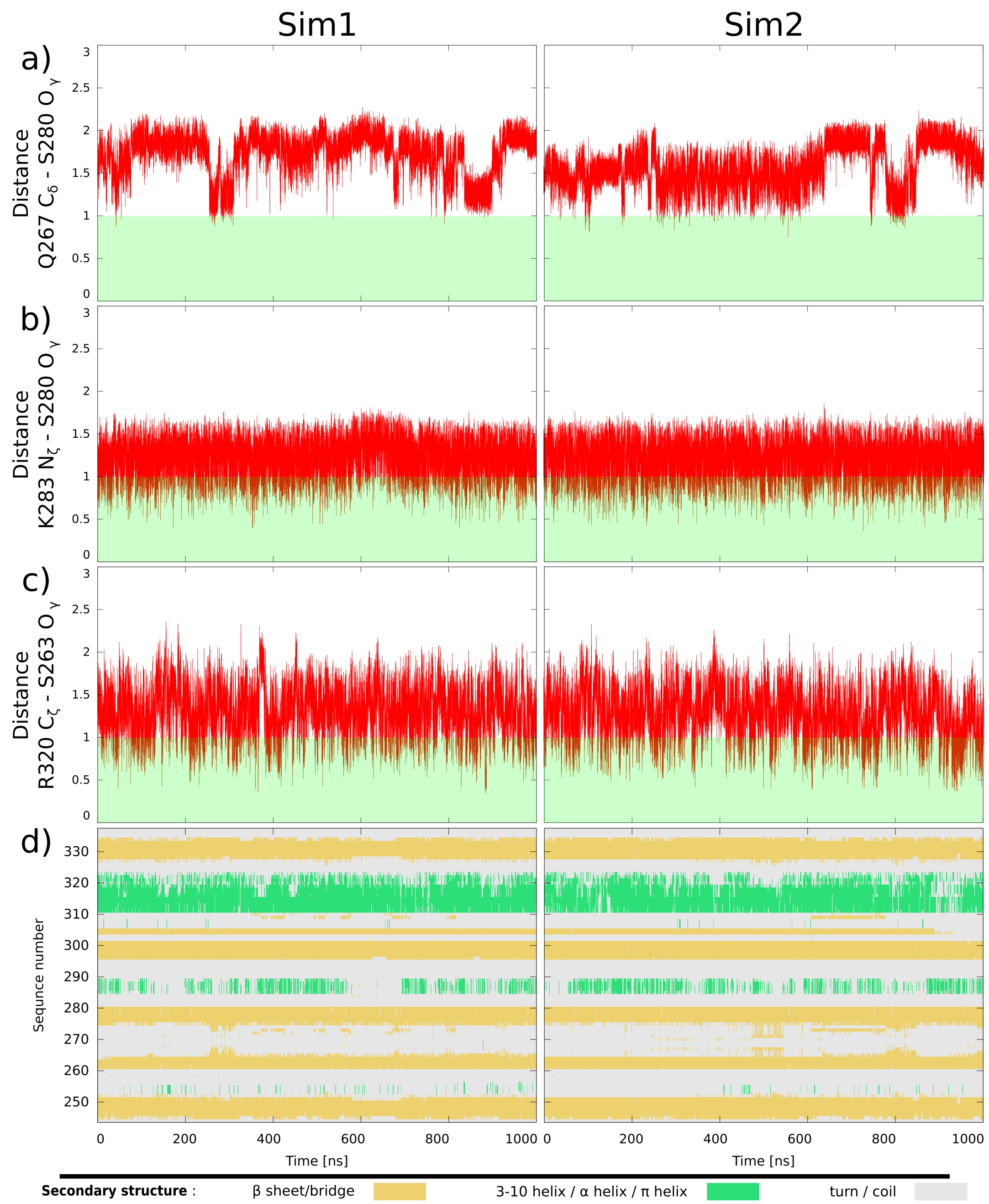

Figure S3. Development of two separate PDZ wt reference simulations. a-c) time dependencies of the distance between particular atoms. d) reference secondary structure development (color coding: below the picture).

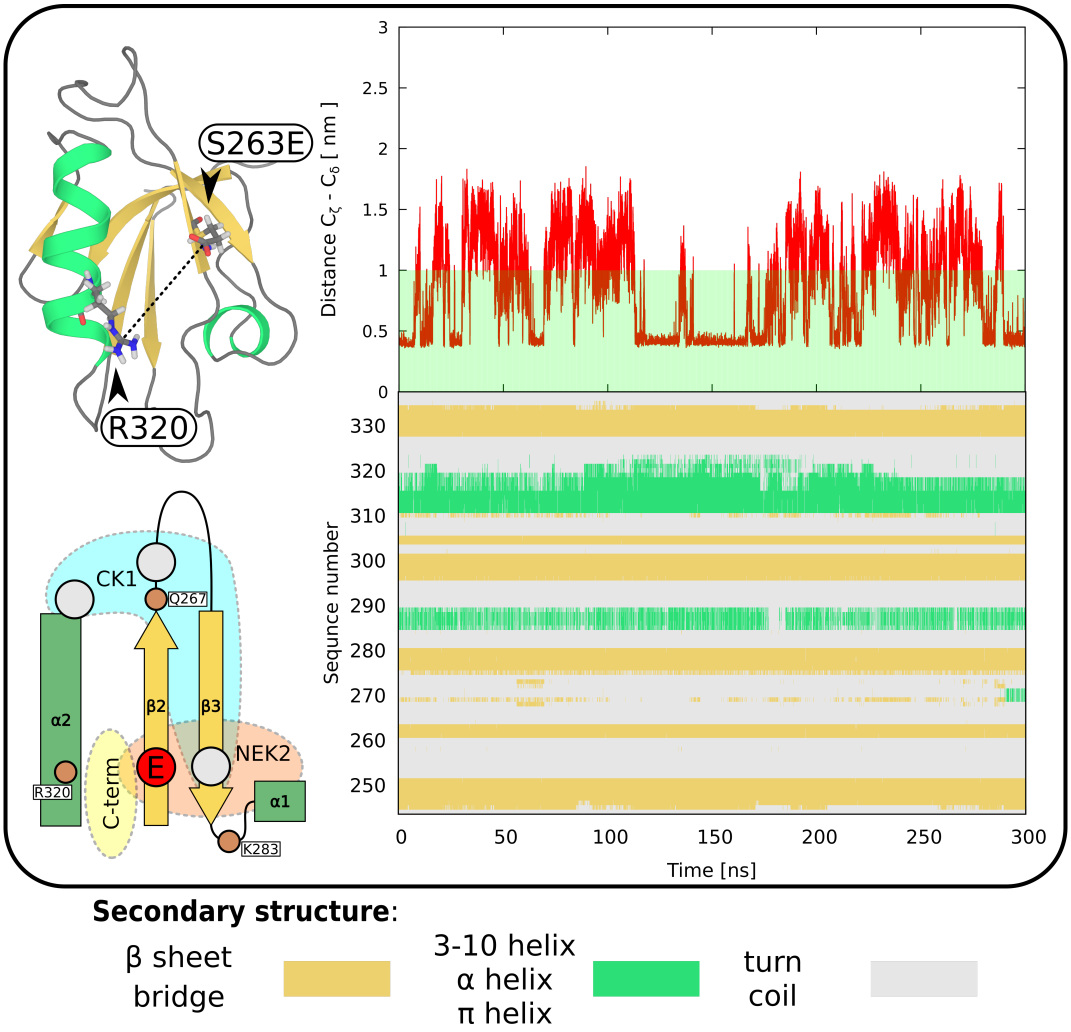

Figure S4. Effects of the phospho mimetic S263E on the PDZ structure where the initial conformation was taken from last snapshot of the pS263. Top left - system snapshot; bottom left - schematic representation; top right - time evolution of the distance between C_ζ atom in R320 and C_δ atom in S263E, respectively; and bottom right - the time evolution of the PDZ secondary structure (color coding: below the picture).

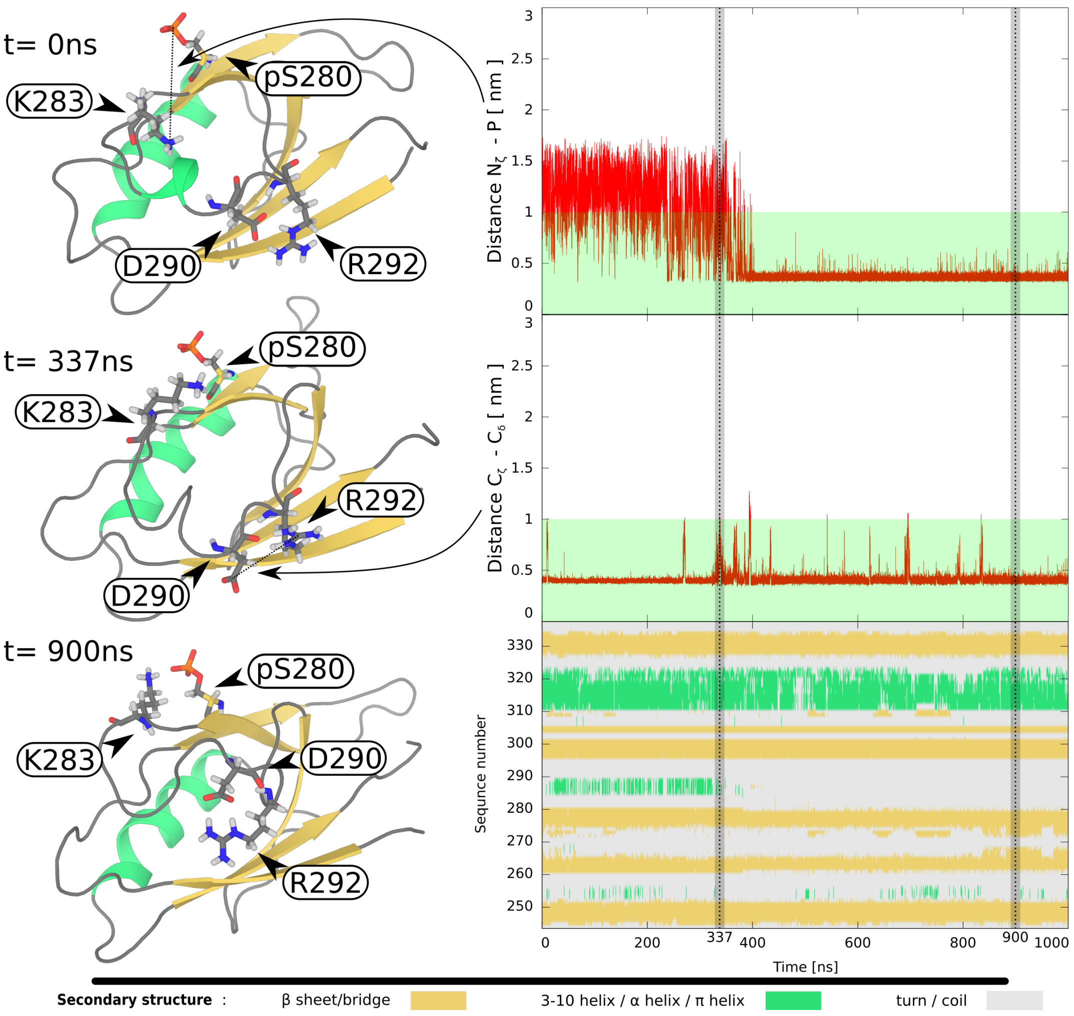

Figure S5. The helix $\alpha 1$ unfolding mechanism. At time t=0 ns the helix $\alpha 1$ is formed and stabilized by the interaction between D290 and R292. At time t=337 ns the interaction between D290 and R292 breaks and enable the helix unfolding. Once the helix $\alpha 1$ unfolded the K283 in the loop preceding the helix $\alpha 1$ forms interaction with pS280 which stabilize the helix in the unfolded state. Afterward the interaction between D290 and R292 reforms and further stabilize the helix in an unfolded state (t=900 ns).

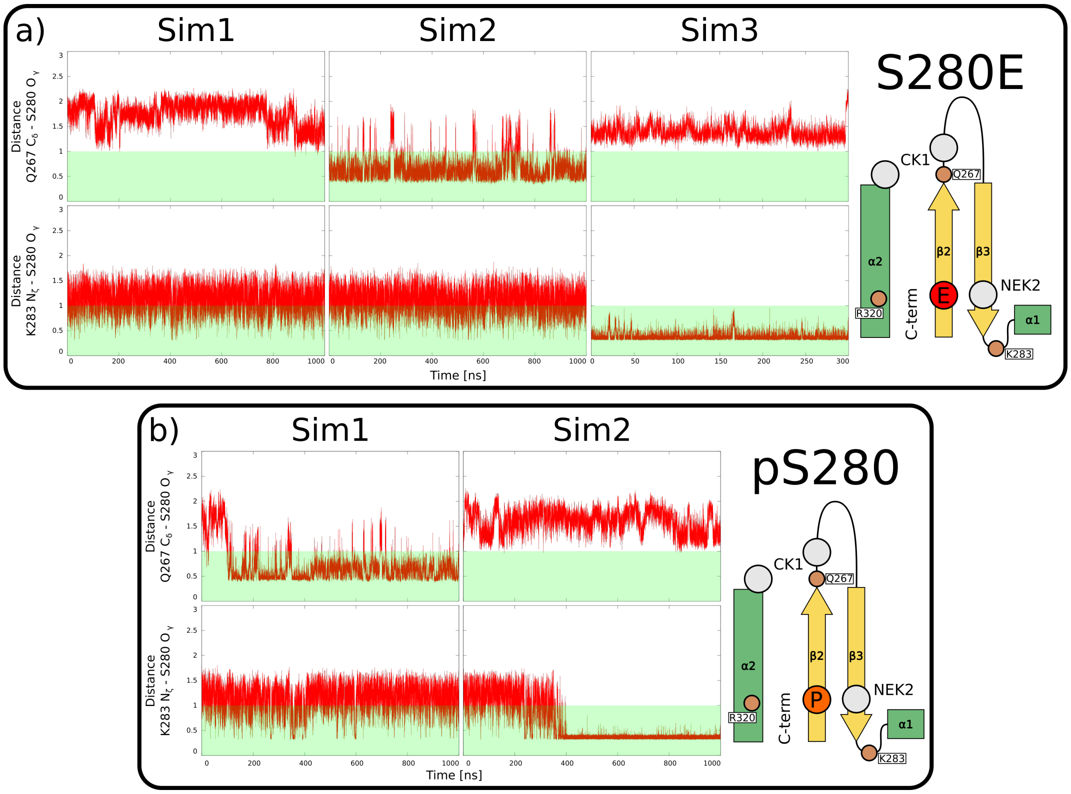

Figure S6. Comparison of two separate simulations of phospho and mimetic variants. a) mimetic and b) phospho variants of S280. Each column corresponds to one of the two simulations. Each first line then corresponds to the distance between Q267 and S280, while each second line corresponds to distance between K283 and S280.

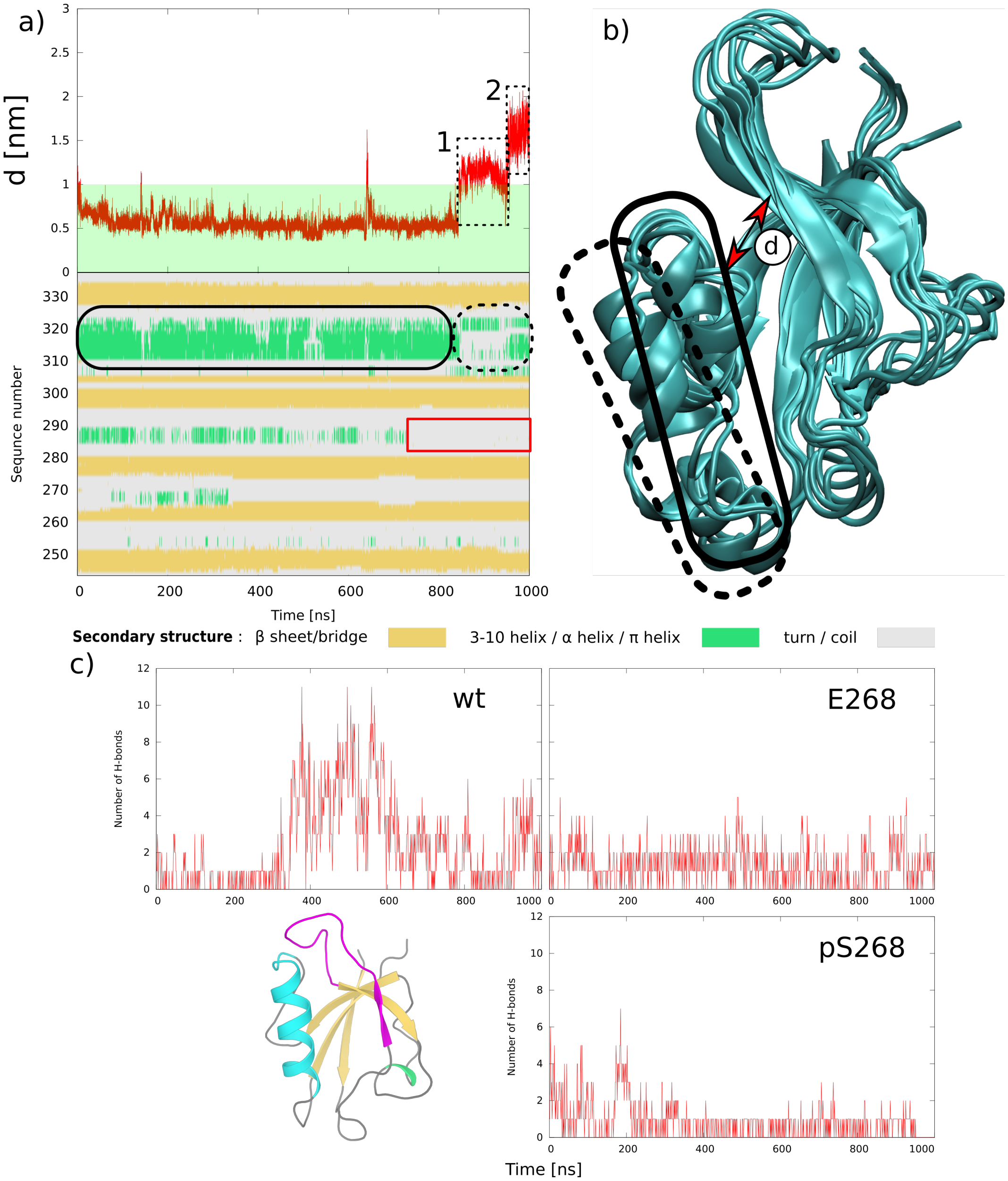

Figure S7. The sliding of the helix $\alpha 2$. a) Time evolution of distance (d) between CA atoms of G266 and N312 which describe the helix $\alpha 2$ shift. Small rectangles correspond to the initial helix shift and central disruption while the second corresponds to the helix refolding in a new position. Below is secondary structure evolution in the simulation of pS268. Unfolding of the short helix $\alpha 1$ followed by unfolding of the helix $\alpha 2$ are highlighted by red rectangles. b) Snapshots from last 100 ns of the 1 μ long simulation every 20 ns. Displacement and the partially unfolded helix $\alpha 2$ are visible if we compare the initial helix position (solid rectangle) to final position (dashed rectangle). c) A number of hydrogen bonds between residues in the loop region and strand (262-275 purple in the figure) and residues in at the N-terminal region of the helix $\alpha 2$ and the helix $\alpha 2$ (309-324 cyan in the figure).

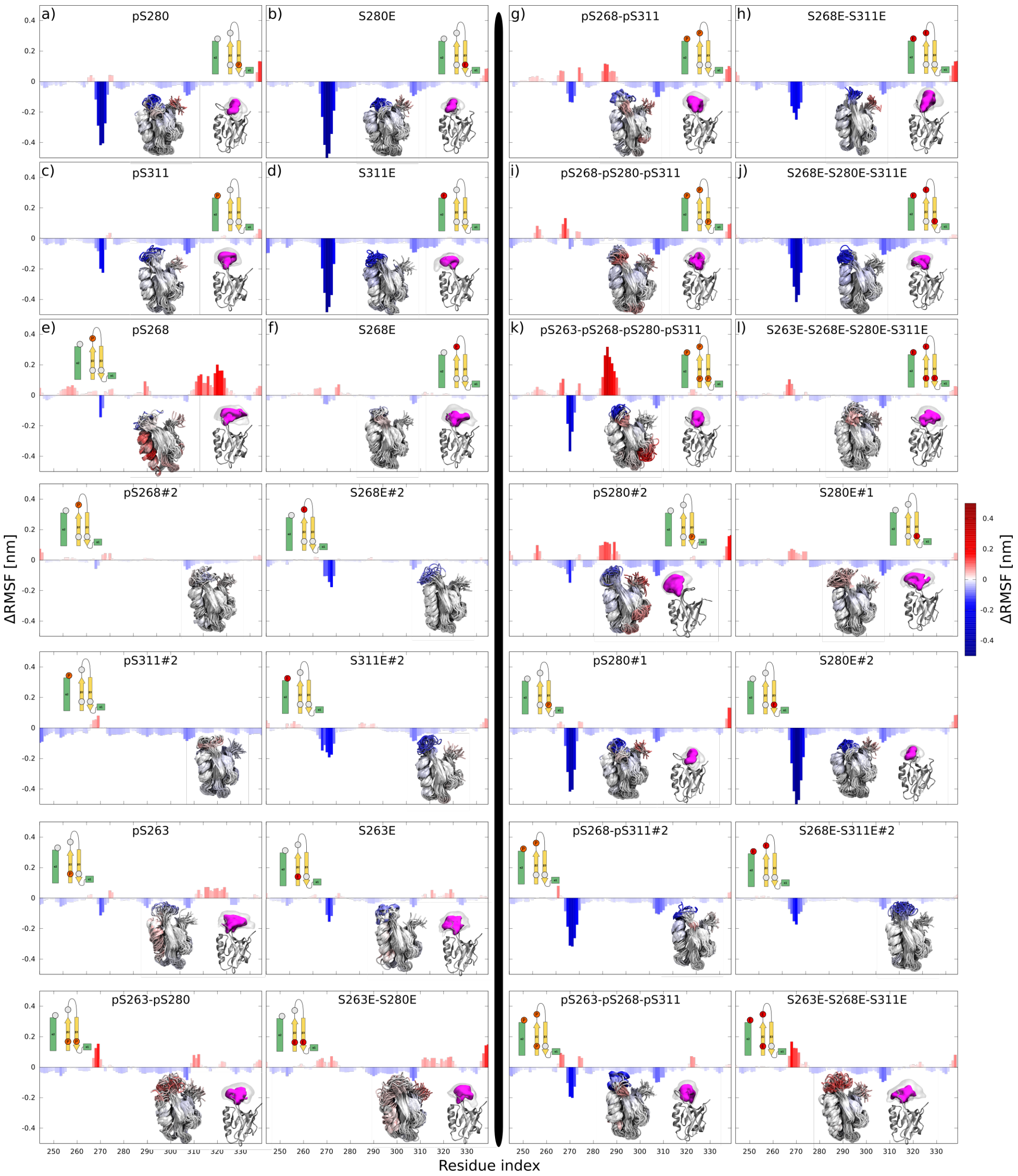

Figure S8. Changes in fluctuations (ΔRMSF) in phospho/mimetic variants with respect to PDZ wt. Positive values (red) and negative values (blue) corresponds to an increase and decrease in RMSF compare to PDZ wt, respectively. In inserts, we have a bundle of simulation snapshots colored according to RMSF change (bottom left), a picture of the volume occupied by $\beta 2$-$\beta 3$ loop (for color coding see Fig 5)(bottom right) and schematic representation of the system according to Fig 1(top). In each pair of graphs on the left is phospho variant while on the right is mimetic variant. In all systems '#' denotes the number of simulation, thus if the system is simulated twice with different initial conditions the second simulation would be denoted '#2'. Note that Simulations with S280 variants are group base on the occurrence of interaction with long loop $\beta 2$-$\beta 3$ which stabilize the loop.

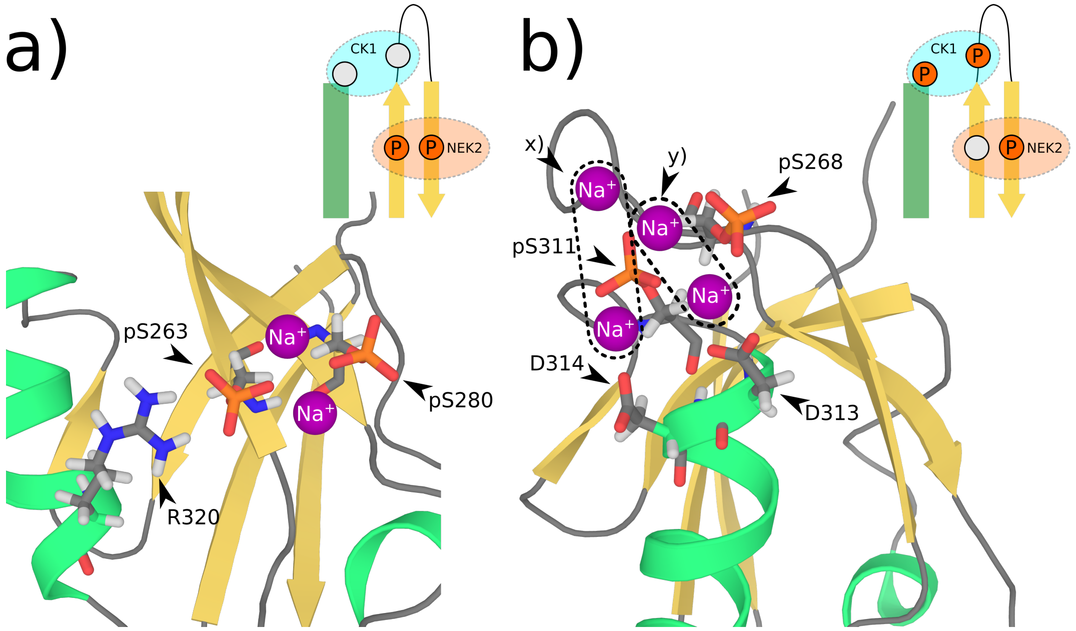

Figure S9. Interaction between two phosphate groups in a) pS263-pS280 and b) pS268-pS280-pS311 are shown. In double phosphorylated PDZ, both sodium ions are stably positioned in between phosphate groups. In triple phosphorylated PDZ x) denote semi-stable ions positioned in between two phosphate groups, while y) more diffuse ion positions that further neutralize phosphate charge and mediated interaction with negatively charged residues in surroundings.

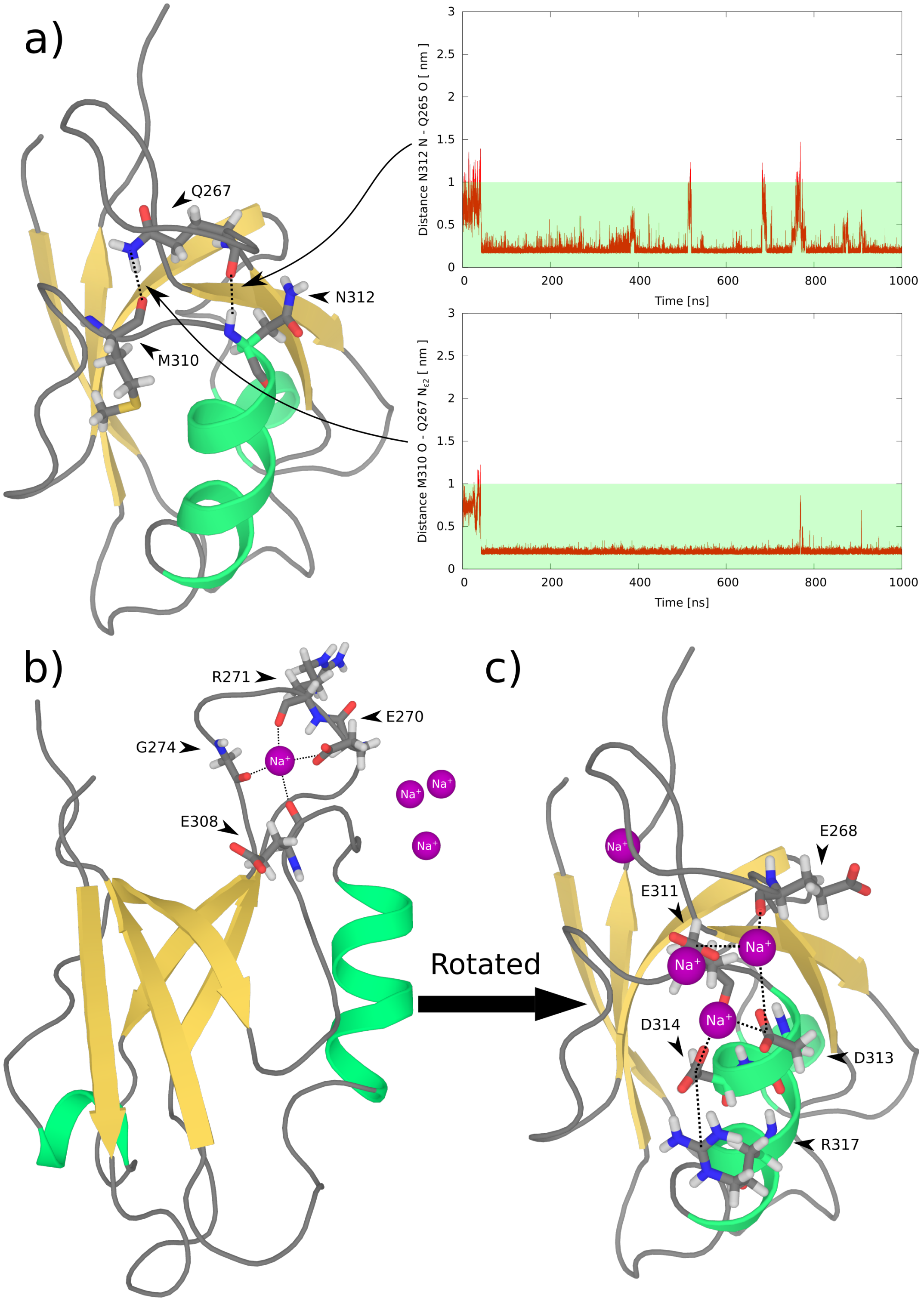

Figure S10. Loop conformation in triple mimetic mutant S268E S280E S311E. a) two stable hydrogen bonds between loop residue Q267 and residues preceding the helix $\alpha 2$ which keep the loop in close proximity to the helix. b) back view of the loop conformation with one central sodium ion interacting with backbone oxygen atoms (G274 and R271) in the loop and glutamic acid side chain (E270) from the loop. c) rotated the view of the loop conformation with three sodium ions that bridge interaction between negatively charged side chains depicted.

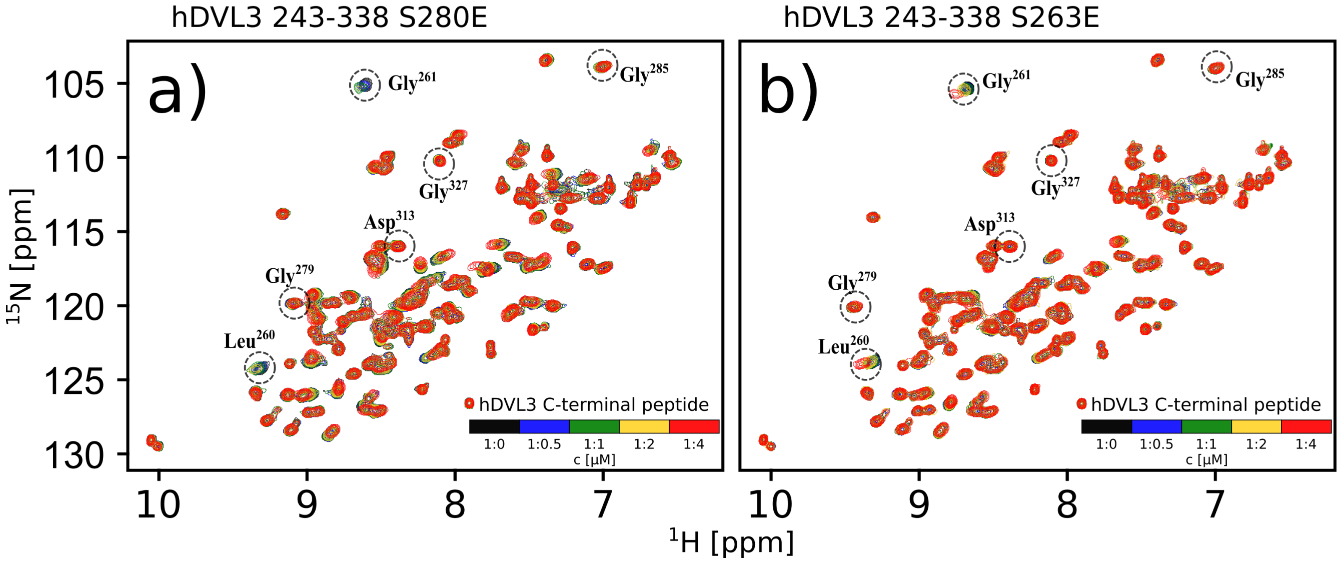

Figure S11. The full spectra of ^{1}H-^{15}N HSQC overlay for S280E and S263E Spectra with increasing concentration of the C-terminal peptide are colored from black (no peptide) to red (four-time higher concentration of peptide with respect to PDZ). The full spectra corresponding to a) S280E mutant and b) S263E mutant.

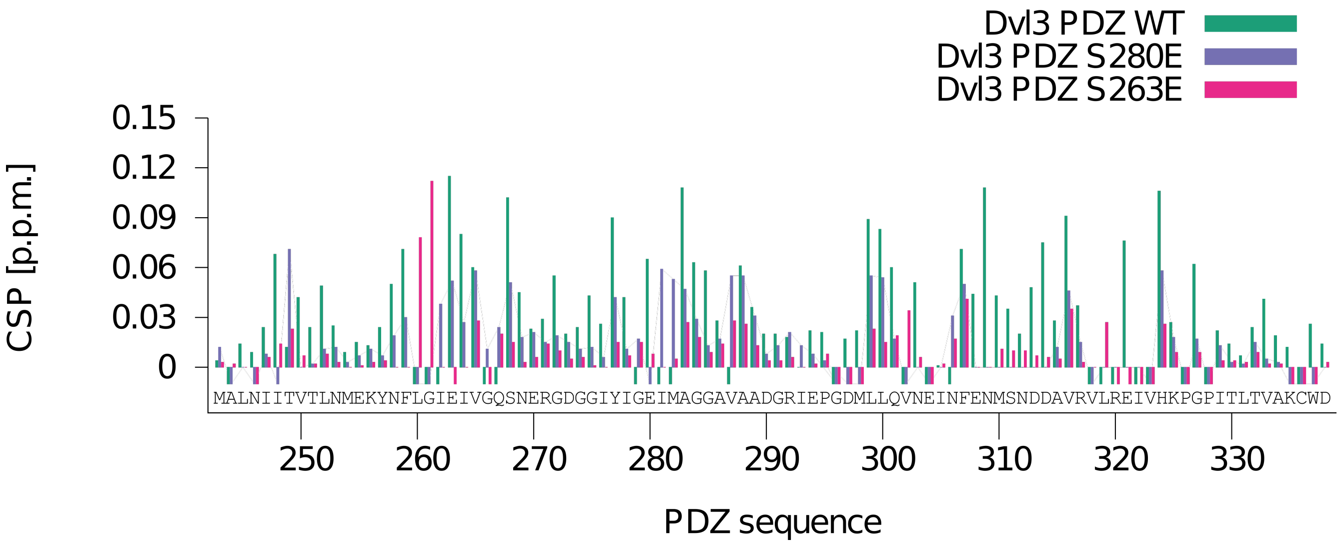

Figure S12. NMR analysis of ligand binding. Weighted chemical shift perturbations (CSPs) between free PDZ wt or phosphomimicking mutants in the presence of four times excess of peptide ligand versus the protein sequence. CSPs that could not be analyzed because of peak overlap or linewidth exchange broadening beyond detection are depicted as negative bars.

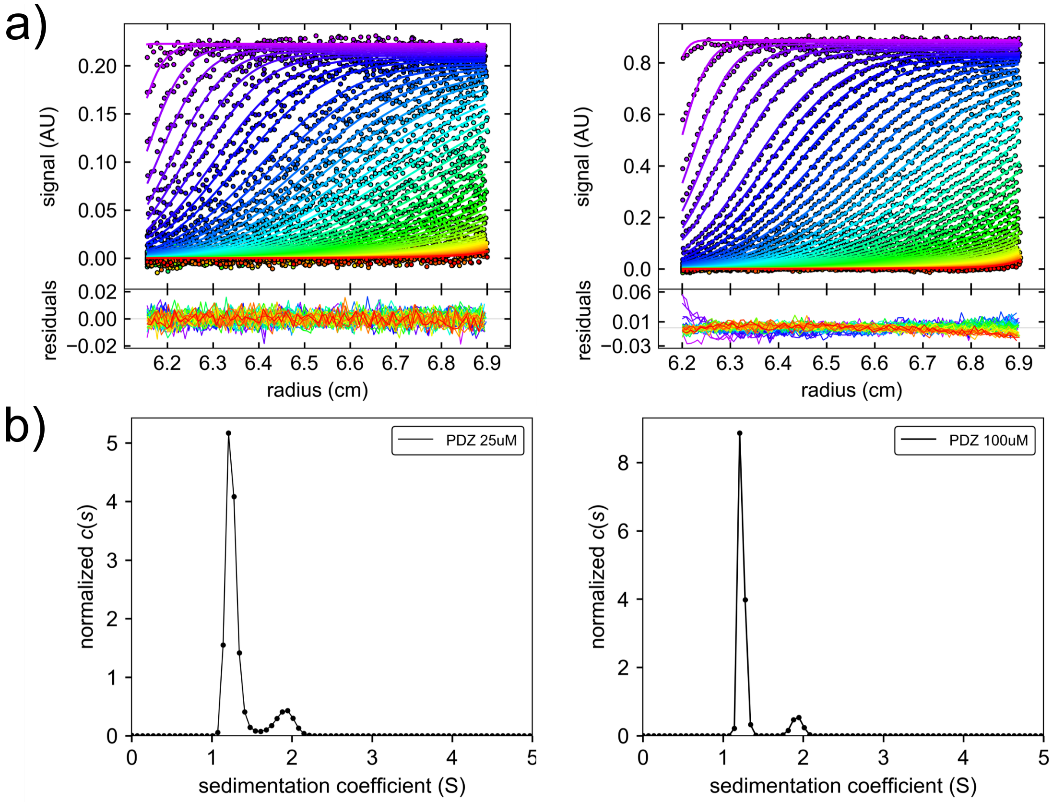

Figure S13. The measurements of sedimentation velocity of the PDZ domain by analytical ultracentrifugation. a) Sedimentation profiles of absorbance at 280 nm for 25 μM (left panels) and 100 μM solution of PDZ (right panels). The sedimentation scans were coloured base on the time of acquisition from violet over blue and green to red. The solid lines represent the best fit to the Lamm equation supplied by SEDFIT software. Residual plot supplied by SEDFIT software is in the bottom. b) The continuous distribution of sedimentation coefficient, c(s), obtained with a regularization procedure. The PDZ was found to be mostly in monomeric form, particularly PDZ monomers accounted for 87.5 and 90.0% at 25 μM and 100 μM PDZ concentration, respectively.

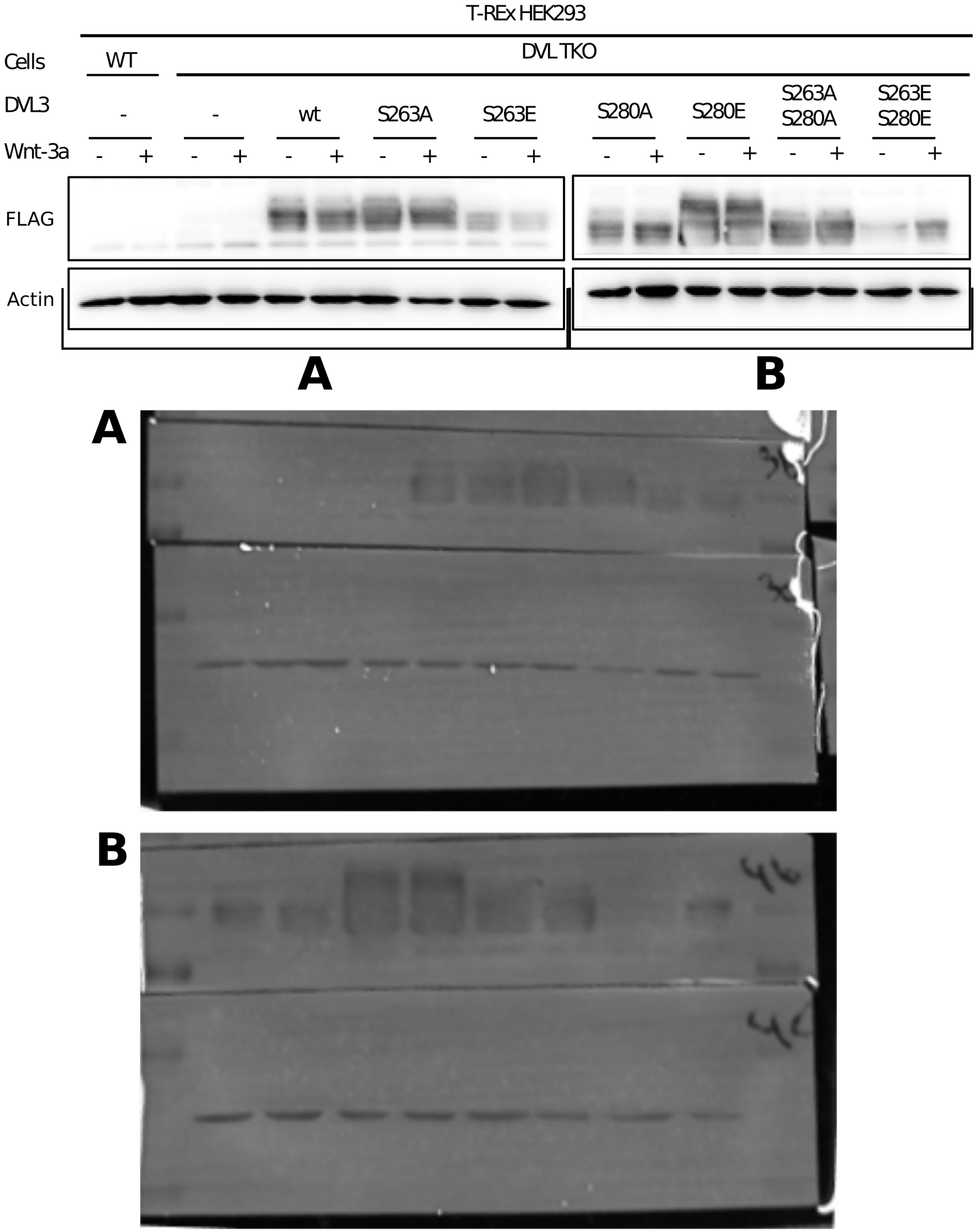

Figure S14. Western blot analysis of expression levels of individual mutants. Original gels are at the bottom. The capital letters relate two parts of cropped gels with their original gels.
